# Evaluating Price and Availability of Essential Medicines in China: A Mixed Cross-Sectional and Longitudinal Study

**DOI:** 10.3389/fphar.2020.602421

**Published:** 2020-11-26

**Authors:** Caijun Yang, Shuchen Hu, Dan Ye, Minghuan Jiang, Zaheer-Ud-Din Babar, Yu Fang

**Affiliations:** ^1^ Department of Pharmacy Administration and Clinical Pharmacy, School of Pharmacy, Xi’an Jiaotong University, Xi’an, China; ^2^ Center for Drug Safety and Policy Research, Xi’an Jiaotong University, Xi’an, China; ^3^ Department of Pharmacy, Xi’an No. 3 Hospital, The Affiliated Hospital of Northwest University, Xi’an, China; ^4^ Center for Pharmaceutical Policy and Practice Research, Department of Pharmacy, University of Huddersfield, Huddersfield, United Kingdom

**Keywords:** medicine price, pharmaceutical policy, equitability, China, availability

## Abstract

**Objectives:** To evaluate the price and availability of medicines in China.

**Methods:** A standard methodology developed by WHO and Health Action International was used to collect medicine price and availability data. We obtained cross-sectional data for 48 medicines from 519 facilities (280 public hospitals and 239 private retail pharmacies) in five provinces in China in 2018. We also collected longitudinal data for 31 medicines in Shaanxi Province in 2010, 2012, 2014, and 2018. Medicine price was compared with the international reference price to obtain a median price ratio (MPR). The availability and price in five provinces were compared in matched sets. We used general estimating equations to calculate differences in availability and median prices from 2010 to 2018.

**Findings:** Mean availability of surveyed medicines in five provinces was low in both public (4.29–32.87%) and private sectors (13.50–43.75%). The MPR for lowest priced generics (LPGs) was acceptable (1.80–3.02) and for originator brands (OBs) was much higher (9.14–12.65). The variation was significant for both availability and price of medicines across provinces. In Shaanxi Province, the availability of medicines decreased between 2010 and 2018, but this was not significant in the public or private sector. Compared with 2010, the median adjusted patient price was significantly lower in 2018 for nine OBs (difference −22.4%; *p* = 0.005) and 20 LPGs (−20.5%; *p* = 0.046) in the public sector and 10 OBs (−10.2%; *p* = 0.047) in the private sector.

**Conclusion:** Access to medicines was found to be poor and unequal across China in 2018. Future interventions are needed, and possible strategies include effective and efficient procurement, promoting the development of retail pharmacies and increasing medicine price transparency.

## Introduction

Access to affordable medicines is essential to achieve universal health coverage (Hogerzeil et al., 2013). However, access is a global concern. Providing full and affordable access to health became a great challenge to all health systems because of the high price of medicines and drug shortage problems (Eom et al., 2016; Burrone et al., 2019).

In China, access to healthcare has been diminished since the market-based reform in the healthcare sector in the late 1970s (Yip and Hsiao, 2008). The high cost of medical products is regarded as a major barrier during that period. In 2008, drug expenditure constituted 41% of total health expenditures (CNHDRC, 2019), whereas the average percentage in OECD countries was only 16% (NHSA, 2019).

To reduce drug expenditure and improve access to affordable medicines, the National Essential Medicine Program was introduced as one of the key pillars of the major healthcare reform initiated in 2009 by the Chinese government (Yip et al., 2019). The core of the program is the National Essential Medicine List (Yip et al., 2012). The first version of the National Essential Medicine List issued in August 2009 contained 307 generic medicines (NHCPRC, 2009). To meet local needs, provinces were allowed to supplement the list in the first few years of their launch (Fang et al., 2013).

In the first stage of the National Essential Medicine Program (from 2009 to 2011), a series of supporting policies were implemented targeting medicine pricing, procurement, prescribing, and reimbursement. A province-based competitive-bidding system for medicines was established to obtain low prices with assured quality. The central government set price ceilings for essential medicines, and manufacturers bid in different provinces through the Internet. Medicines were procured for the whole province at agreed bid prices. All public primary healthcare institutions were required to stock and use essential medicines and to sell them at cost (referred to as zero-markup drug policy). In secondary and tertiary hospitals, the essential medicine use targets were set by the provincial health department and varied across provinces. To improve the use of essential medicines, local social health insurance programs provided higher reimbursement for essential medicines than nonessential medicines (Yip et al., 2012).

Based on the early experience and evaluation results of the National Essential Medicine Program, the pharmaceutical policy has evolved quickly in the following years. The government started to mandate the zero-markup drug policy for county-level hospitals in 2012 and for city-level hospitals in 2015. In the second half-year of 2017, all public hospitals had implemented zero-markup drug policy. In 2014, primary healthcare sites were allowed to procure and prescribe a small number of nonessential medicines (NHCPRC, 2014). In 2015, the government price ceiling was eliminated. Starting from the second half of 2015, the bidding procedure was abolished for specific formulary of medicines (Hu et al., 2019). The second and third editions of the National Essential Medicine List were issued in 2012 and 2018, with 520 and 685 generics, respectively. Major policies are described in Supplementary Appendix S1.

Ten years have passed since the initiation of the National Essential Medicine Program. Although medicine price and availability decreased in several provinces in the early years after the implementation of the National Essential Medicine Program (Fang et al., 2013; Liu et al., 2014; Xi et al., 2015), little is known about their current patterns. This information is essential to develop interventions or informing necessary midcourse adjustments that aim to improve access to medicines nationally in China. Accordingly, we measured medicine prices and availability by a cross-sectional survey in a sample of public hospitals and private retail pharmacies in five provinces in 2018 and with a longitudinal survey in a single province from 2010 to 2018.

## Methods

### Study Design

We undertook a cross-sectional survey of medicine availability and prices in China from December 2017 to January 2018, with a standard methodology developed by World Health Organization and Health Action International (WHO/HAI) (Health Action International, 2008).

Five out of 31 provincial-level regions in mainland China were selected, including Shandong, Hubei, Henan, Shaanxi, and Yunnan. These provinces were purposely selected to cover diverse characteristics of geographical location (eastern, central, and western China) and socioeconomic status (GDP per capita, high, middle, and low). More details regarding the sampling of the national cross-sectional survey are provided in Supplementary Appendix S2.

In each province, six municipal regions stratified by socioeconomic status were randomly selected. Within each province, we first stratified the municipal regions into three strata according to the GDP per capita ranking from highest to lowest. Then, we randomly selected two municipal regions from each stratum (here, the provincial capital city was selected at first). In each area, the main government hospital (tertiary hospital) was included and five other government hospitals were randomly selected (from those within a 4 h drive from the main hospital). A private pharmacy near each selected public health facility was also surveyed. When the availability of the surveyed medicine was less than 50% at a given outlet (except the main hospital), the corresponding nearest outlet would be surveyed as a backup. The distribution and the number of all facilities sampled were listed in Table 1.

**TABLE 1:** Distribution and number of sample facilities.

Area	Public hospitals				Private pharmacies	Total facilities
	Primary	Secondary	Tertiary	Total		
Shandong (subtotal)	28	13	6	47	46	**93**
Qingdao	5	2	1	8	9	17
Jinan	4	2	1	7	7	14
Rizhao	6	3	1	10	8	18
Weifang	5	2	1	8	7	15
Jining	4	2	1	7	7	14
Linyi	4	2	1	7	8	15
Hubei (subtotal)	34	22	6	62	58	**120**
Wuhan	6	4	1	11	11	22
Yichang	6	3	1	10	8	18
Xiaogan	5	4	1	10	9	19
Xianning	6	4	1	11	11	22
Suizhou	5	4	1	10	9	19
Enshi	6	3	1	10	10	20
Henan (subtotal)	33	15	6	54	44	**98**
Zhengzhou	6	2	1	9	6	15
Jiaozuo	6	2	1	9	7	16
Luohe	5	2	1	8	10	18
Xinxiang	6	4	1	11	8	19
Nanyang	5	3	1	9	7	16
Zhoukou	5	2	1	8	6	14
Shaanxi (subtotal)	36	22	6	64	55	**119**
Yulin	6	2	1	9	10	19
Xi’an	6	4	1	11	7	18
Baoji	6	4	1	11	12	23
Xianyang	6	4	1	11	9	20
Weinan	6	4	1	11	9	20
Shangluo	6	4	1	11	8	19
Yunnan (subtotal)	35	12	6	53	36	**89**
Kunming	6	2	1	9	6	15
Yuxi	6	2	1	9	6	15
Qujing	6	2	1	9	6	15
Honghe	5	2	1	8	6	14
Pu’er	6	2	1	9	6	15
Wenshan	6	2	1	9	6	15
Total	166	84	30	280	239	519

Among a total of 48 medicines included in the survey (Supplementary Appendix S3), 12 belong to the core list suggested by WHO/HAI (referred to as global list), and 36 were added as supplementary medicines. The supplementary list was prepared on the basis of local disease burden and local needs and then finalized after feedback from international experts (from WHO/HAI) and an advisory group of practicing pharmacists, academics, and experts from five provincial centers for medicine procurement.

As required by the WHO/HAI method, data were collected during on-site visits to each facility. For each medicine, the data of price (charged to patients) and availability of originator brand (OB) and lowest-price generic equivalent (LPG) were collected. The LPG for each medicine was determined at the facility level. The data collecting method was standard suggested by WHO/HAI and can refer to other published works (Fang et al., 2013).

Additionally, we have already undertaken three cross-sectional surveys of medicine availability and prices in Shaanxi Province in 2010, 2012, and 2014 previously. Shaanxi Province is ranked midlevel in GDP per capita among the 31 provincial regions in China and is broadly representative of the typical health and health system status in China. The same cities were selected for the survey in four years, where 31 medicines were found in common (details in Supplementary Appendix S4).

### Data Quality Control

The project manager was responsible for the whole survey. In each province, one expert from the school of pharmacy at local university or local hospital pharmacy was in charge of the provincial survey, namely, the provincial survey manager. To gather and record accurate and reliable data, several data quality assurance processes were implemented throughout the study.

Initially, before the survey, firstly, materials introducing the survey and survey procedure were designed according to the WHO/HAI workbook and were sent to the provincial survey manager in September 2017. Secondly, a training workshop was conducted by researchers in November 2017. The provincial survey manager and one pharmacy student or pharmacist (appointed as the associate provincial survey manager) who would go to the site personally in each province took the training workshop. In this phase, attendees were trained about the data collection process, completing the data collection form and data entry. In addition, all attendees rehearsed with researchers who were familiar with the WHO/HAI method in a private pharmacy and a hospital pharmacy. Third, each provincial manager was required to train all the data collectors in his/her province, following the same procedure and using the same materials provided by the project manager.

During the survey, the provincial survey manager was responsible for the quality control of the provincial survey and was required to check the data at the end of each survey day. The surveys were started before the end of January 2018 and were finished in one month. The accurate start time in each province was decided by the provincial survey manager.

After the survey, the data collected on the medicine price data collection form were entered by designated personnel into the software International Medicines Price Workbook (2015). The prices were double entered to ensure accuracy. The workbook’s auto checker was used to assist in the verification process. Finally, the provincial survey manager sent all data collection forms and the electronic workbooks to the project manager. Among all the data, 10% of the collection forms were rechecked by the project manager.

### Statistical Analysis

Availability in each province is reported as the percentage of outlets in which each medicine was found on the day of data collection. Most WHO/HAI surveys use the criteria of availability of medicines as follows: very low: < 30%, low: 30–49%, fairly high: 50–80%, high: > 80% (Sharma et al., 2016; Kibira et al., 2017; Liu et al., 2017; Gong et al., 2018; Sun et al., 2018; Wu et al., 2018; Kasonde et al., 2019). Median unit prices (MUPs) for each medicine is calculated in RMB (yuan, ¥). The median price for each medicine type in each sector is calculated only if the medicine was available in at least three public or private facilities in each province. Medicine prices were compared with the international reference price (IRP) to obtain a median price ratio (MPR). The IRPs are the medians of recent procurement or tender prices offered by predominantly not-for-profit suppliers to developing countries for multisource products (Management Science for Health, 2015). Normally, an MPR of one or less is taken as efficient procurement in the public sector, while below three, it is considered efficient for the private sector (Babar et al., 2007). Here, MPR of three or less is considered an acceptable price for both the public and private sectors.

The availability of 48 medicines in the five provinces was compared in matched sets. The medicines found available in all five provinces were included for price analysis. The MPR for each medicine was calculated and then compared among the five provinces for matched sets. Friedma’s test was applied.

For the longitudinal survey in Shaanxi, we used generalized estimating equations to test differences in the availability of 31 medicines included in the four surveys, with facility specified as a random effect. To compare the unit price in the four years in Shaanxi, we adjusted the 2018, 2014, and 2012 unit prices to 2010 prices by deflating them by CPI of 20.68%, 13.71%, 8.66%, respectively (National Bureau of Statistics, 2020). We used the Friedman test to identify whether the reductions or increases in adjusted median prices between 2010 and 2018 were significant. To examine the effect of surveyed outlets, using the same methods, sensitivity analysis of a subset of outlets that included in all rounds was conducted. We took *p* < 0.05 as a significant difference in all statistical testing.

## Results

### National Survey

#### Availability

**FIGURE 1:**
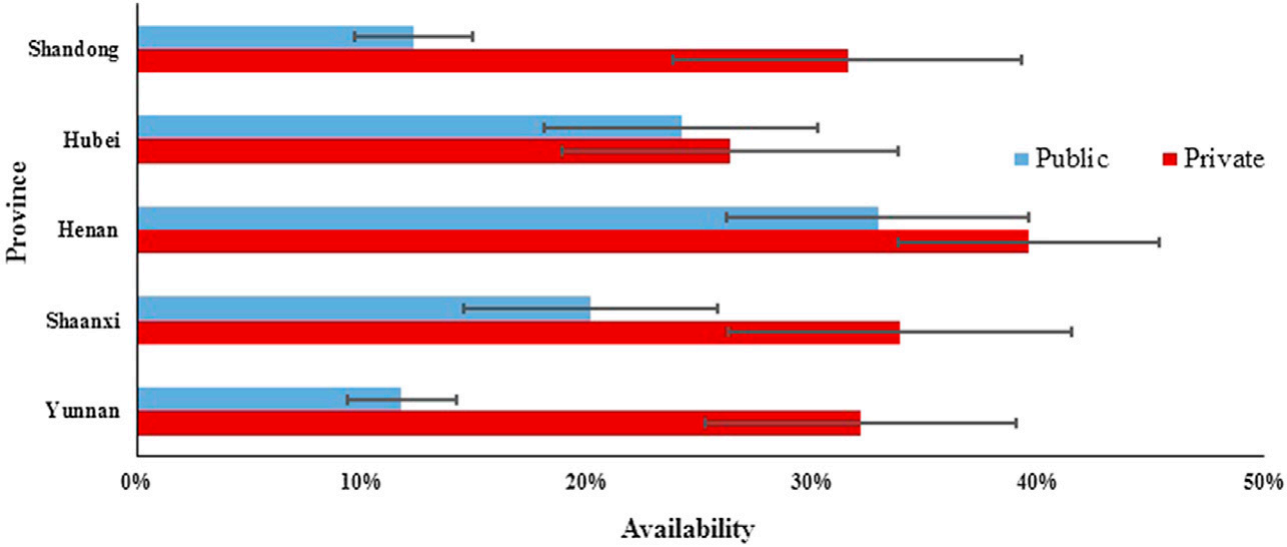
The availability of generics in public and private sectors in five provinces.

**FIGURE 2:**
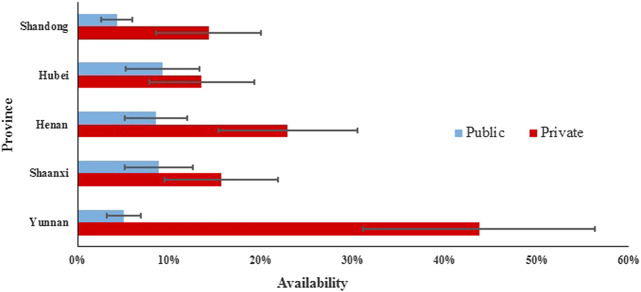
The availability of OBs in public and private sectors in five provinces.

In the public sector of five provinces, the mean availability of 48 surveyed medicines ranged from 11.75% to 32.87% for generics and from 4.29 to 9.27% for OBs. For generics in five provinces, the mean availability of medicines on the NEML and medicines for acute disorders was numerically higher than medicines not on the NEML and medicines for chronic disorders, respectively, whereas opposite results were noted for OBs. Additionally, there was a significant difference in mean availability of 48 OBs (*p* = 0.001) and 48 generics (*p* < 0.001) across provinces. Of all medicines, Henan, Shaanxi, and Hubei had higher mean availability than Shandong and Yunnan for both OBs and LPGs. For LPGs, the provincial difference was significant for all subgroups of medicines (NEMLs and non-NEML medicines; global and supplementary medicines; medicines for acute and chronic disorders), whereas, for OBs, the difference was only significant for 38 medicines on NEML, 36 supplementary medicines, and 28 medicines for chronic disorders (Table 2, Figures 1, 2).

**TABLE 2:** Availability of 48 medicines in public sector hospitals and private retail pharmacies in five provinces.

	Shandong	Hubei	Henan	Shaanxi	Yunnan		*p*
Mean availability of generics in the public sector (%)							
All medicines (48)	12.27	24.16	32.87	20.15	11.75	＜ 0.001	
NEML (38)	13.64	27.59	36.70	21.75	12.83	＜ 0.001	
Non-NEML (10)	7.08	11.13	18.33	14.06	7.67	0.001	
Global (12)	11.87	28.09	37.65	22.79	12.15	＜ 0.001	
Supplementary (36)	12.41	22.85	31.28	19.27	11.62	＜ 0.001	
Acute (20)	13.25	25.56	36.20	23.75	13.29	＜ 0.001	
Chronic (28)	11.58	23.16	30.49	17.58	10.65	＜ 0.001	
Mean availability of OBs in public sector (%)							
All medicines (48)	4.29	9.27	8.53	8.85	5.00		0.001
NEML (38)	3.99	8.28	7.55	7.52	4.43		0.003
Non-NEML (10)	5.42	13.06	12.22	13.91	7.16		0.258
Global (12)	5.76	9.94	9.41	11.59	5.90		0.144
Supplementary (36)	3.80	9.05	8.23	7.94	4.70		0.003
Acute (20)	2.37	4.52	4.17	4.14	2.33		0.160
Chronic (28)	5.65	12.67	11.64	12.22	6.90		0.003
Mean availability of generics in the private sector (%)							
All medicines (48)	31.52	26.29	39.54	33.83	32.12	＜ 0.001	
NEML (38)	33.12	27.68	41.93	33.88	32.16	＜ 0.001	
Non-NEML (10)	25.44	21.03	30.46	33.64	31.95	0.044	
Global (12)	33.88	31.18	39.58	36.97	32.18	0.743	
Supplementary (36)	30.74	24.66	39.52	32.78	32.10	＜ 0.001	
Acute (20)	35.33	28.10	41.14	33.82	34.31	0.026	
Chronic (28)	28.80	25.00	38.39	33.83	30.56	＜ 0.001	
Mean availability of OBs in the private sector (%)							
All medicines (48)	14.27	13.50	22.87	15.64	43.75	＜ 0.001	
NEML (38)	13.96	12.70	20.75	14.16	36.04	＜ 0.001	
Non-NEML (10)	15.44	16.55	30.91	21.27	73.05	0.001	
Global (12)	16.85	15.37	26.14	18.94	45.14	0.076	
Supplementary (36)	13.41	12.88	21.78	14.55	43.29	＜ 0.001	
Acute (20)	9.56	10.17	18.41	9.9	35.14	＜ 0.001	
Chronic (28)	17.62	15.89	26.05	19.74	49.90	＜ 0.001	

NEML, 2012 National Essential Medicines List; LPG, lowest-price generic; OB, originator brand.

In the private sector of five provinces, the mean availability of 48 surveyed medicines ranged from 26.29% to 39.54% for generics and from 13.50 to 43.75% for OBs. For generics, the mean availability of medicines on the NEML, medicines on the global list, and medicines for acute disorders was higher than medicines not on the NEML, supplementary medicines, and medicines for chronic disorders, respectively. For OBs, the mean availability of medicines not on the NEML, medicines on the global list, and medicines for chronic disorders was higher than medicines on NEML, supplementary medicines, and medicines for acute disorders. Similarly, there was a significant difference in the mean availability of 48 OBs (*p* < 0.001) and 48 generics (*p* < 0.001) across the provinces. For generics, the provincial difference was only significant for 38 medicines on NEML, 36 supplementary medicines, and 28 medicines for chronic disorders, whereas, for OBs, the difference was significant for all subgroups of medicines except the 12 medicines on the global list.

For individual medicines, the availability varied by type of medicine, sector, and province (Supplementary Appendix S5). The private sector had higher availability than the public sector for both generics and OBs in all five provinces. For both public and private sectors in the five provinces, OBs for chronic disorders had higher availability than that for acute disorders, while for generics, opposite results were noted except the private sector in Shaanxi Province where the generics for acute and chronic disorders had similar availability. For generics in the public sector, Shandong and Yunnan had no medicine with the availability of over 50%, while Henan, Hubei, and Shaanxi had 10, 8, and 6 medicines with at least 50% availability, respectively. For generics in the private sector, the number of medicines with at least 50% availability ranged from 10 to 15 in the five provinces; among these medicines, six (azithromycin, enalapril, glimepiride, loratadine, metronidazole, and omeprazole) were common in five provinces. For OBs in the public sector, only Hubei had one medicine (albendazole) with the availability of 50% and other provinces had none. In Yunnan Province for OBs in the private sector, 23 products had availability over 50%, whereas other four provinces had three to eight medicines with the availability of at least 50%. Among these medicines, formulations of two medicines (albendazole and atorvastatin) were common in five provinces.

#### Medicine Prices

There are 32 LPGs and 16 OBs included in price analysis in terms of MPRs (Table 3). The median price of glargine analog insulin (without IRP) is provided in Supplementary Appendix S6, which shows that OB in the private sector has a higher price than that in the public sector; however, the price of LPG in the two sectors has no significant difference.

**TABLE 3:** Median of MPRs in public and private sectors in five provinces.

	Shandong	Hubei	Henan	Shaanxi	Yunnan	*p*
Median of MPR for LPGs in the public sector						
All medicines (33)	2.24	1.96	1.85	1.80	2.55	0.080
NEML (26)	2.43	1.59	2.17	1.75	2.38	0.193
Non-NEML (7)	2.01	1.97	1.72	2.57	2.69	0.038
Global (9)	1.93	1.97	2.62	2.62	2.85	0.918
Supplementary (24)	2.90	1.93	1.36	1.53	1.96	0.062
Acute (14)	0.91	1.08	1.19	1.07	1.54	0.576
Chronic (19)	3.57	2.47	2.74	3.29	2.85	0.041
Median of MPR for OBs in the public sector						
All medicines (15)	12.65	11.92	12.64	12.45	10.22	0.062
NEML (12)	13.42	12.79	15.68	13.13	11.43	0.154
Non-NEML (3)	6.94	7.45	7.61	7.41	9.96	0.229
Global (7)	8.34	8.38	8.82	8.54	9.96	0.099
Supplementary (8)	13.42	12.89	15.68	13.14	11.69	0.075
Acute (5)	19.80	18.26	19.51	18.57	19.80	0.452
Chronic (10)	9.68	10.15	9.92	10.49	8.07	0.155
Median of MPR for LPGs in the private sector						
All medicines (32)	2.28	2.66	1.66	2.82	3.02	0.001
NEML (26)	2.28	2.77	1.61	3.01	2.84	0.039
Non-NEML (6)	2.69	2.24	1.78	2.80	3.84	0.007
Global (9)	2.87	2.85	1.89	3.24	3.73	0.036
Supplementary (23)	2.05	2.55	1.58	2.45	2.73	0.020
Acute (8)	2.24	2.74	1.33	2.59	2.80	0.103
Chronic (18)	2.49	2.66	2.08	3.05	3.89	< 0.001
Median of MPR for OBs in the private sector						
All medicines (17)	9.23	9.27	9.14	9.24	9.39	0.030
NEML (13)	13.09	12.45	10.10	12.45	11.57	0.129
Non-NEML (4)	6.96	6.12	6.51	6.13	7.29	0.090
Global (6)	7.88	7.25	7.11	7.17	7.81	0.505
Supplementary (11)	12.67	11.87	10.10	12.31	11.57	0.076
Acute (5)	16.85	13.83	16.85	16.85	14.26	0.187
Chronic (12)	8.34	8.39	8.49	8.72	8.50	0.077

Data are median unit price (number of products found in all five provinces). NEML, 2012 National Essential Medicines List; LPG, lowest-price generic; OB, originator brand.

In the public sector, the MPRs for LPGs and OBs across provinces ranged from 1.80 to 2.55 and from 10.22 to 12.65, respectively. There was no significant difference in MPRs across provinces, LPGs (*p* = 0.08) or OBs (*p* = 0.062), while for the seven non-NEML LPGs and 19 LPGs for chronic disorders, a significant difference was noted in MPRs across provinces. The LPGs for chronic disorders have higher MPRs than that for acute disorders, while an opposite situation was observed for OBs.

In the private sector, the MPRs for LPGs and OBs ranged from 1.66 to 3.02 and from 9.14 to 9.39, respectively. A significant difference was noted in MPRs for both LPGs (*p* = 0.001) and OBs (*p* = 0.030) across provinces. For LPGs, the provincial differences were significant for all subgroups of medicines except medicines for acute disorders, whereas, for OBs, no significant difference among provinces was found when considering the six groups of medicines (NEML and non-NEML medicines; global and supplementary medicines; medicines for acute and chronic disorders).

Comparatively, the median MPRs for LPGs in the public sector are lower than that in the private sector (except Henan Province); the opposite is noted for OBs. Compared with the other four provinces, Yunnan Province had the highest median MPR for LPGs and OBs in the private sector, LPGs in the private sector, and lowest median MPR for OBs in the public sector.

### Longitudinal Survey

The number of facilities surveyed in Shaanxi Province varied in the four years; data from 20 public hospitals included in all rounds were used for sensitivity analysis (Supplementary Appendixes S7, S8).

#### Availability

**FIGURE 3:**
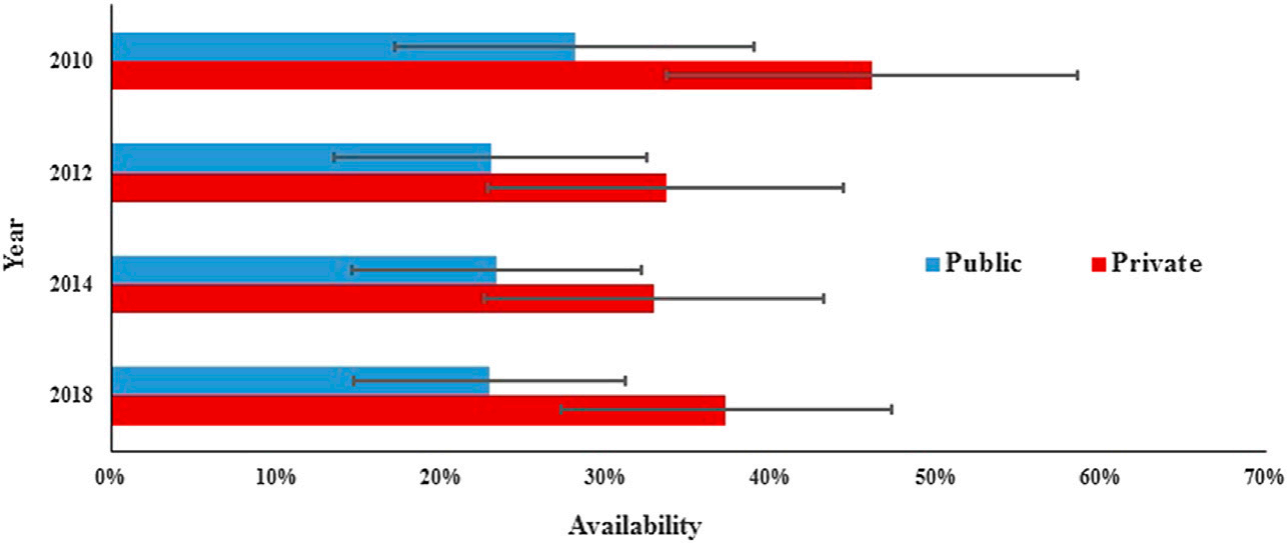
The availability of generics in public and private sectors in Shaanxi Province.

**FIGURE 4:**
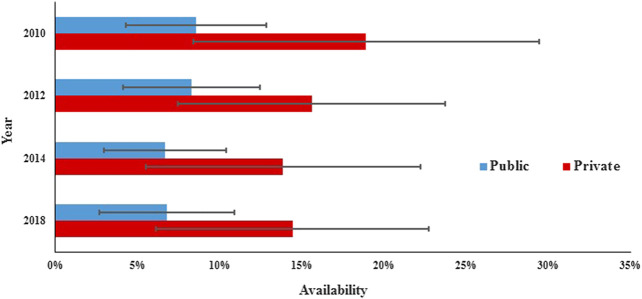
The availability of OBs in public and private sectors in Shaanxi Province.

In the public sector, the availability of surveyed medicines was poor in the four rounds. Compared with 2010 (28.1%), there was a significant decrease in mean availability for 31 generics in 2012 (23.0%; *p* = 0.000) and 2014 (23.3%; *p* = 0.017) and no significant change in 2018 (23.0%; *p* = 0.073), and similar results were found for the generics listed on NEML and supplementary list. The mean availability for 31 OBs has decreased continuously from 2010 to 2018, but the decrease was not statistically significant (8.6% vs. 8.3%, 7.6%, and 6.8%; *p* = 0.679, 0.082, and 0.267, respectively). Similar results were noted for all subgroups of medicines, except medicines on the global list) (Table 4, Figures 3, 4).

**TABLE 4:** Availability of 31 medicines in public hospitals and private retail pharmacies in Shaanxi Province.

	Mean availability in the public sector (%)				Mean availability in the private sector (%)			
	2010	2012	2014	2018	2010	2012	2014	2018
Generics								
All medicines	28.1	23.0	23.8	23.0	46.1	38.8	32.9	37.3
NEML (25)	33.2	26.6	26.3	26.8	26.9	26.6	18.1	25.5
Non-NEML (6)	7.0	8.1	13.0	7.0	50.8	41.8	36.4	40.1
Global (11)	30.6	22.7	23.2	23.6	49.7	40.3	29.8	36.2
Supplementary (20)	26.8	23.2	24.1	22.7	44.2	38.1	34.6	37.9
Acute (15)	29.7	23.8	22.7	25.8	46.7	41.2	33.0	37.9
Chronic (16)	26.6	22.3	24.7	20.3	45.7	36.6	32.8	36.7
OBs								
All medicines	8.6	8.3	7.6	6.8	18.9	15.6	13.8	14.4
NEML (25)	8.4	8.6	7.9	7.3	18.1	15.1	15.0	14.8
Non-NEML (6)	9.3	7.2	6.1	4.7	22.2	17.8	9.0	12.7
Global (11)	9.8	11.2	11.9	11.8	15.7	15.4	15.7	18.0
Supplementary (20)	7.9	6.7	5.2	4.1	20.7	15.7	12.8	12.5
Acute (15)	8.1	7.4	6.2	5.0	21.1	15.8	11.9	13.2
Chronic (16)	9.0	9.1	8.8	8.5	16.8	15.4	15.6	15.6

NEML, 2012 National Essential Medicines List; LPG, lowest-price generic; OB, originator brand.

In the private sector, the availability of survey medicines in the four years was higher than that in the public sector; however, they were still low. Compared with 2010 (46.1%), there was a significant decrease in mean availability for 31 generics in 2012 (38.8%; *p* = 0.047), 2014 (32.9%; *p* = 0.000), and 2018 (37.3%; *p* = 0.013). Excluding generics listed on the global list and NEML, the availability of the other four subgroups of generics in 2018 has no significant difference with that in 2010. Compared with 2010 (18.9%), there was no significant change in mean availability for 31 OBs in 2012 (15.6%, *p* = 0.066), 2014 (13.8%, *p* = 0.071), and 2018 (14.4%, *p* = 0.169); similar results were noted for all the six subgroups of OBs.

#### Medicine Prices

In the public sector, the median price charged to patients continued decreasing from 2010 to 2018 for OBs, whereas the patient price for LPGs decreased in 2012 and 2018 but increased in 2014. The median percentage decrease in prices for all 20 common LPGs between 2010 and 2018 was 20.5% (IQR 39.3 to −16.9; *p* = 0.046), while for nine OBs, the decreases was 22.4% (IQR 20.0–34.8; *p* = 0.005) (Table 5).

**TABLE 5:** Median price of 31 medicines in public hospitals and private retail pharmacies in Shaanxi Province.

	2010	2012	2014	2018	Changes from 2010 to 2018
Median price of LPGs in the public sector (RMB)					
All medicines (20)	0.37	0.33	0.44	0.24	−20.5 [−39.3, 16.9]^*^
NEML (17)	0.28	0.15	0.25	0.18	−6.8 [−37.2, 20.7]
Non-NEML (3)	1.30	0.96	0.82	0.81	−36.2 [−51.9, −27.9]
Global (7)	0.47	0.41	0.42	0.17	−26.5 [−55.7, 1.1]^*^
Supplementary (13)	0.28	0.26	0.47	0.50	−21.4 [−36.2, 20.7]
Acute (9)	0.47	0.41	0.50	0.22	−33.5 [−47.9, 20.7]
Chronic (11)	0.16	0.15	0.15	0.50	−6.8 [−31.4, 12.3]
Median price of OBs in public sector (RMB)					
All medicines (9)	4.60	3.29	3.20	3.17	−22.4 [−34.8, −20.0]^*^
NEML (8)	4.25	3.18	3.12	3.09	−22.5 [−35.7, −19.7]^*^
Non-NEML (1)	—	—	—	—	—
Global (5)	4.6	3.3	3.0	3.0	−34.8 [−38.5, −22.6]^*^
Supplementary (4)	4.9	4.0	4.0	3.9	−19.3 [−20.2, 17.4]^*^
Acute (4)	9.1	8.1	7.5	7.1	−20.6 [−26.7, −17.4]^*^
Chronic (5)	4.6	3.3	3.0	3.0	−22.4 [−34.8, −21.0]^*^
Median price of LPGs in the private sector (RMB)					
All medicines (23)	0.50	0.46	0.47	0.55	11.4 [−14.8, 55.5]
NEML (19)	0.24	0.17	0.38	0.50	30.0 [−1.8, 84.3]
Non-NEML (4)	1.71	0.73	1.11	1.28	−17.1 [−23.7, −8.2]
Global (9)	0.50	0.15	0.38	0.54	11.4 [8.7, 30.0]^*^
Supplementary (14)	0.77	0.61	0.77	0.91	21.5 [−15.1, 57.8]
Acute (10)	0.59	0.42	0.52	0.55	20.7 [−9.0, 57.8]
Chronic (13)	0.18	0.52	0.16	0.86	9.6 [−14.2, 50.0]
Median price of OBs in the private sector (RMB)					
All medicines (10)	4.57	3.05	3.46	3.97	−10.2 [−13.3, −4.7]^*^
NEML (8)	3.33	3.05	2.89	3.25	−11.5 [−13.8, 0]
Non-NEML (2)	—	—	—	—	—
Global (4)	2.59	2.21	1.99	2.23	−12.6 [−16.4, −8.7]
Supplementary (6)	5.86	3.77	4.63	5.17	−7.6 [−12.5, 9.9]
Acute (4)	8.04	7.01	6.57	7.44	4.9 [−6.9, 22.8]
Chronic (6)	4.57	2.77	3.45	3.93	−12.0 [−14.8, −10.1]^*^

NEML, 2012 National Essential Medicines List; LPG, lowest-price generic; OB, originator brand. * Friedman test p < 0.05.

In the private sector, the median retail price decreased in 2012 and then started increasing for both LPGs and OBs. Compared with 2010, in 2018, the median price increased 11.4% for 23 LPGs (IQR −14.8 to 55.5; *p* = 0.657) and decreased 10.2% for 10 OBs (IQR −13.3 to −4.7; *p* = 0.047).

The LPGs for chronic disorders have lower MPRs than that for acute disorders in the public sector during 2010–2014, while the situation was changed in 2018 and the MPRs for chronic disorders became higher than that for acute disorders, whereas the OBs for acute disorders have higher MPRs than that for chronic disorders all the time in both public and private sectors.

## Discussion

This study revealed that there were several deficiencies in the availability and prices of medicines in China. First, the mean availability of surveyed medicines in all five provinces was regarded as low or very low in both public and private sectors according to the criteria recommended by most WHO/HAI surveys. Second, the prices of LPGs were acceptable, but OBs were expensive. Third, there was unequal availability and price of medicines among different provinces. Fourth, the medicine price kept decreasing since 2010, especially for OBs in the public sector, while there was no significant change in the availability between 2010 and 2018.

The availability of surveyed medicines was insufficient for both sectors, while comparing with the public hospitals, private pharmacies usually have higher mean availability, higher price for LPGs, and lower price for OBs. Most previous studies of this type have been in developing countries. Comparatively, the availability of LPGs in our study was similar to the results in Pakistan (Saeed et al., 2019) and Philippines (Lambojon et al., 2020) but lower than those in Bangladesh (Kasonde et al., 2019), Malawi (Khuluza and Haefele-Abah, 2019), Jordan (Alefan et al., 2018), and Rwanda (Bizimana et al., 2020), where the availability of LPGs was fairly high; however, the availability of OBs in those countries was similar. Comparing with the price in other developing countries, LPGs in our study had similar or a little higher price, while OBs had a much higher price. The reasons for gaps in the availability and prices of medications in China are not clear. As reported by a prior WHO/HAI study, poor medicine availability can be due to a combination of factors including inadequate funding, inability to forecast accurately, lack of incentives for maintaining stocks, and inefficient distribution systems (Cameron et al., 2009). Whatever, the low availability and high price of medicine, which would diminish access to medicines and increase the economic burden for patients, are of great concern.

This study adds to the scientific literature in several ways: it is the first study of the availability and cost of medications in China using the standard WHO/HAI method involving multiple provinces. Previously, similar studies focused on a single province and were carried out at different time periods separately which impedes provincial comparison (Yang et al., 2010; Jiang et al., 2013; Wang et al., 2014; Jiang et al., 2015; Xi et al., 2015; Gong et al., 2016; Li et al., 2019; Zhu et al., 2019). This study provides a comparative assessment of the availability and price of medicines across provinces and shows that large variances exist. Moreover, most studies were implemented in the first few years after 2009, while a lot of national pharmaceutical policies aiming to improve access were released in recent years. This study includes longitudinal data which tracks the changing process of availability and price of medicines in China.

We found unequal availability and prices of medicines across provinces. In the public sector, a significant variance was observed in availability but not in price, and province-level centralized bidding and purchasing system may partly contribute to this result. The pharmaceutical firms and suppliers were selected through a competitive-bidding process by the provincial committee’s jurisdiction. Usually, the provincial committee set price cap for a medicine, referring to other provinces’ prices of the same product provided by the same manufacturer. In the private sector, a variance was observed in both availability and prices of medicines, and the reason is not clear. Lack of regulations on prices may contribute to the price differences in the private pharmacies (Gong et al., 2018). The development of the retail pharmacy industry in each province also may affect the availability and price of medicines in the private sector (Yang et al., 2010). The literature has also suggested that regional disparity in health resources’ allocation and health service utilization might be due to the gaps in economic level and transport system among different regions (McLeod et al., 2011; He et al., 2013; Sun and Luo, 2017; Guan et al., 2018). However, we only included five provinces in the survey and this impacts on detecting the relationship of availability and price of medicine with economic level or transport system.

We found a dynamic change process of availability and price of medicines from 2010 to 2018 in Shaanxi Province. Compared with 2010, the availability decreased in 2012, but the decrease slowed in the following years and for some medicines, the availability even increased in 2018. On the other hand, the price of medicines decreased significantly in 2018 except LPGs in the private sector, when compared with the 2010 prices. This result might be due to a series of national-level policies particularly since 2014 aiming to ensure medicine supply and the zero-markup drug policy implementation in all public hospitals. However, as the policy was only implemented at the provincial level, whether Shaanxi’s research findings can be generalized to other provinces of China is not clear.

The implications of this study for medication policies in China are substantial. The poor availability of medicines and the high price of OBs demonstrated that more effective policies are needed to be implemented to improve patients’ access to medicines. The decreasing trend of availability of medicines was curbed in Shaanxi Province and the price of LPGs was acceptable. Fortunately, in the late 2018s, China adopted a novel procurement scheme named a nationwide collective pharmaceutical procurement, which significantly decreased the prices of selected medicines. Twenty-five medicines were awarded contracts to manufacturers in the pilot program and the average price decreasing by 52% (National Healthcare Security Administration, 2018). Pooling the demands of member cities could be a good option to increase the bargaining power on price negotiation (Jiang et al., 2020). Additionally, as stated by our study, the availability of LPGs for chronic diseases was lower than that for acute diseases, while the contrary was observed for OBs. It was observed that the prices of OBs were much higher than that of generic medicines. Hence, increasing the availability of generic medicines for chronic diseases would largely decrease the economic burden for China, as shown from the literature that the chronic diseases could contribute toward poverty in China (Lan et al., 2018).

The study has also other important implications. Access to essential medicines is a vital component of fulfilling the right to health, while a lack of equity in the supply of essential medicines impedes its realization (United Nations, 1948). Equitable access to medicines is a fundamental component for progress in achieving universal health coverage, and it is one of Sustainable Development Goals of the United Nations (Wirtz et al., 2017). Healthy China 2030 also includes justice and equity as one of four core principles (Tan et al., 2017). Our study showed that unequal access to medicines occurs across provinces, especially in price variation in the private sector. This shows that effective and efficient procurement policies and interventions promoting the development of retail pharmacies are needed. In addition, strategies to increase medicine price transparency in the private sector may lead to increased access to medicines in the long term (Ahmad et al., 2019).

Our study had several limitations. The survey methodology required only including medicines with a specific strength and dosage form. This could result in underestimation of the availability of some medicines, as some facilities may have stocked other dosage forms or strengths of the surveyed medicines on the day of data collection. It means that, for the same medicines, other strengths and dosage forms may be available; however, they were not taken into account while checking the availability and price of medicines. Second, the availability of medicines was low overall, which might make the median unit prices (MUPs) less robust especially for OBs in the public sector. Third, our sample was drawn from five randomly selected provinces and may not be fully representative for the whole of China despite including a large number of randomly selected representative public and private facilities. Fourth, sample facilities were not consistent in the four-round surveys in Shaanxi Province, which constrained our ability to assess the availability and price changes from 2010 to 2018. Nevertheless, sensitivity analysis performed in the subset of common facilities mostly confirms the results.

In conclusion, this study revealed key obstacles in improving equitable access to medicines in China. Measures are needed to improve equitable access to medicines, including effective and efficient procurement policies, promoting the development of retail pharmacies and increasing medicine price transparency in the private sector.

## Data Availability Statement

The original contributions presented in the study are included in the article/supplementary material; further inquiries can be directed to the corresponding author.

## Ethics Statement

The Ethics Committee of Xi’an Jiaotong University Health Science Center (Xi’an, China) reviewed this study and stated that no formal ethics approval was required in this particular case. Oral consents were obtained from all participating organizations.

## Author Contributions

Designed the study: CY, YF. Collected part of the data: CY, MJ. Checked and analyzed the data: CY, SH, DY. Drafted the article: CY. Critical revision of the manuscript: CY, SH, DY, ZB, YF. Approval of the final version of the manuscript: CY, SH, DY, MJ, ZB, YF.

## Funding

This work was supported by National Natural Science Foundation of China (71503197 and 71473192), Department of Science and Technology of Shaanxi Province (2020 SF-279), and “the Fundamental Research Funds for the Central Universities.” The funders of the study had no role in study design, data collection, data analysis, data interpretation, writing of the report, or the decision to submit the manuscript.

## Conflict of Interest

The authors declare that the research was conducted in the absence of any commercial or financial relationships that could be construed as a potential conflict of interest.
