# Supplementary material for: Evaluating Price and Availability of Essential Medicines in China: A Mixed Cross-Sectional and Longitudinal Study
Source: Front Pharmacol. 2020 Nov 26;11:602421. doi: 10.3389/fphar.2020.602421 (PMC7768899; doi:10.3389/fphar.2020.602421)
Supplement: Supplementary file 1 [file DataSheet1_v1.zip › 2. Supporting information-2020-10-2.docx]

**Supporting information**

**Appendix 1. Major policies about essential medications’ availability and affordability since 2009**

The National Essential Medicine Program (NEMP) was introduced in the Chinese health care reform 2009. To support the NEMP, many medicine policies targeting medicine manufacturing, pricing, procurement, prescribing and reimbursement were implemented. In general, it includes: 1) issuing a national essential medicine list, and requiring health care facilities to store and prescribe listed medicines, especially the primary health care facilities; 2) establishing province-based competitive-bidding system, to achieve lowest price while assuring quality; 3) dispensing of essential medicines at zero mark-up; 4) providing higher reimbursement for essential medicines than non-essential medicines. Based on results of early implementation, these policies are dynamically changed and constantly adjusted. The following table shows major policies about essential medications’ availability and affordability since 2009.

| **Year** | **Department** | **Policy** | **Link** |
| --- | --- | --- | --- |
| 2009 | MOH | National Essential Medicine List (2009) | http://www.gov.cn/gongbao/content/2013/content_2421032.htm |
|  | CFDA | Notice on strengthening production and quality monitoring in essential medicines | http://www.hbyxjzcg.cn/policy/show565.html |
|  | MIIT | Notice on strengthening in supply of essential medicines | http://iitb.hainan.gov.cn/iitb/zwdt/200910/7f44dd019c5548bda375d1dba7935fa2.shtml |
|  | 5 Ministries^1^ | Working specification in medicine procurement | http://www.nhc.gov.cn/yaozs/s3573/200906/9a461c1cd4e74a08ac80014edfcb0d26.shtml |
| 2010 | State council | The guidance for establishing and normalizing the medicine procurement mechanism among government-sponsored primary health care sites | http://www.gov.cn/gongbao/content/2010/content_1765276.htm |
| 2012 | MOH | National Essential Medicine List (2012) | http://www.gov.cn/gzdt/2013-03/15/content_2355142.htm |
|  | MIIT; MOH;  NDRC; CFDA | Fixed-point production in medicines with small dosage but essential for clinical treatment | http://www.hbyxjzcg.cn/policy/show542.html |
| 2014 | 8 Ministries^2^ | Opinion on ensuring the supply of the commonly-used low-cost medicines | http://www.nhc.gov.cn/yaozs/s3573/201404/900c9f1fbe954acb80f829c0f4d96f3d.shtml |
|  | NHFPC | Opinion on strengthening the storage and use of medicines among primary health care sites | http://www.nhc.gov.cn/yaozs/s3585/201409/0806fb2f25654abc976670d795e792da.shtml |
|  | NHFPC | Notice about managing the procurement of commonly used low priced drugs | http://www.nhc.gov.cn/yaozs/s3573/201406/9b87d64a51094320ad467d7cc6e99f9a.shtml |
| 2015 | 7 Ministries^3^ | Notice about issuing the opinion on promoting drug prices reform | https://www.ndrc.gov.cn/xxgk/zcfb/tz/201505/t20150505_963815.html |
|  | NHFPC; SATCM | Notice about emergency medicines purchasing and supply | http://www.nhc.gov.cn/yaozs/s3573/201501/9c593dfb16e645638d9eab1728c988bc.shtml |
|  | State council | The guidance for public hospital medicine centralized purchasing | http://www.nhc.gov.cn/yaozs/s3577/201502/7d0741e719e249689ec12d62c7936513.shtml |
| 2016 | 8 Ministries^4^ | Issuing the notice about promoting the implementation of the two-invoice drug procurement at public health care facilities | http://www.nhc.gov.cn/tigs/s2906/201701/b64ca4c3d5c64a4c860316437d6eb787.shtml |
|  | NHFPC; MIIT; NDRC;  CFDA | 2016 Fixed-point production in medicines with small dosage, in short supply, but essential for clinical treatment | http://www.gov.cn/xinwen/2016-12/21/content_5151068.htm |
| 2018 | NHC; SATCM | National Essential Medicine List (2018) | http://www.nhc.gov.cn/yaozs/s7656/201810/c18533e22a3940d08d996b588d941631.shtml |

**Abbreviations:**

MOH: Ministry of Health

CFDA: China Food and Drug Administration

MIIT: Ministry of Industry and Information Technology

NDRC: National Development and Reform Commission

NHFPC: National Health and Family Planning Commission

NHC: National Health Committee

MOF: Ministry of Finance

MHRSS: Ministry of Human Resources and Society Security

MOC: Ministry of Commerce

CFDA: China Food and Drug Administration

SAIC: State Administration of Industry and Commerce

SATCM: State Administration of Traditional Chinese Medicine

STA: State Taxation Administration

MRCSC: Medical Reform Office of the State Council

Note:

1. Including: MOH, NDRC, SAIC, CFDA, and SATCM;

2. Including: NHFPC, NDRC, MIIT, MOF, MHRSS, MOC, CFDA, and SATCM;

3. Including: NDRC, NHFPC, MHRSS, MIIT, MOF, MOC, and CFDA;

4. Including: MROSC, NHC, CFDA, NDRC, MIIT, MOC, STA, and SATMC.

**Appendix 2. Sampling of the national cross-sectional survey**

In the mainland China, there are 31 provincial-level regions (provinces, autonomous regions and provincial-level municipality cities, districts). The per capita GDP of these regions (in year 2015) are shown as following table:

| **Provincial-level regions** | **Per capita GDP (Chinese Yuan)** | **Ranking of GDP per capita** |
| --- | --- | --- |
| Tianjin | 107960 | 1 |
| Beijing | 106497 | 2 |
| Shanghai | 103796 | 3 |
| Jiangsu | 87995 | 4 |
| Zhejiang | 77644 | 5 |
| Inner Mongolia | 71101 | 6 |
| Fujian | 67966 | 7 |
| Guangdong | 67503 | 8 |
| Liaoning | 65354 | 9 |
| Shandong | 64168 | 10 |
| Chongqing | 52321 | 11 |
| Jilin | 51086 | 12 |
| Hubei | 50654 | 13 |
| Shaanxi | 47626 | 14 |
| Ningxia | 43805 | 15 |
| Hunan | 42754 | 16 |
| Qinghai | 41252 | 17 |
| Hainan | 40818 | 18 |
| Hebei | 40255 | 19 |
| Xinjiang | 40036 | 20 |
| Heilongjiang | 39462 | 21 |
| Henan | 39123 | 22 |
| Sichuan | 36775 | 23 |
| Jiangxi | 36724 | 24 |
| Anhui | 35997 | 25 |
| Guangxi | 35190 | 26 |
| Shanxi | 34919 | 27 |
| Tibet | 31999 | 28 |
| Guizhou | 29847 | 29 |
| Yunnan | 28806 | 30 |
| Gansu | 26165 | 31 |

Data are from China Statistical Yearbook 2016.

All the 31 provincial-level regions (provinces, autonomous regions and provincial-level municipality cities, districts) are usually divided into eastern, central and western regions based on economic and geographical characteristics. The eastern region includes 11 provinces and municipalities: Beijing, Tianjin, Hebei, Liaoning, Shanghai, Jiangsu, Zhejiang, Fujian, Shandong, Guangdong, and Hainan. The central region includes 8 provinces: Shanxi, Jilin, Heilongjiang, Anhui, Jiangxi, Henan, Hubei, and Hunan. The western region comprises 12 provinces, autonomous regions and municipalities: Inner Mongolia, Chongqing, Guangxi, Sichuan, Guizhou, Yunnan, Tibet, Shaanxi, Gansu, Qinghai, Ningxia, and Xinjiang.

As required by the WHO/HAI methodology, we need survey at least 36 public hospitals and 36 private pharmacies located in 6 different areas to be representative for one province or one region. Therefore, we excluded three provincial-level municipality cities (Beijing, Shanghai and Tianjing) and one province (Hainan) with limited geographical coverage. Furthermore, five autonomous regions (Xinjiang, Tibet, Inner Mongolia, Guangxi, and Ningxia) were also not included as they usually use ethno medicines. Finally, five provinces (Shandong, Hubei, Henan, Shaanxi and Yunnan) were selected for final survey.

In each province, six municipal regions stratified by socioeconomic status were randomly selected. Within each province, we first stratified the municipal regions into three strata according to the GDP per capita ranking from highest to lowest. Then we randomly selected two municipal regions from each stratum (here, the provincial capital city was selected at first). The ranking of socioeconomic status of each municipal region in surveyed provinces were shown as the following tables:

| **Ranking of socioeconomic status of each municipal region in Shaanxi Province** | | | | | | |
| --- | --- | --- | --- | --- | --- | --- |
| Rank of GDP per capita | Municipal regions | Gross domestic products (billions Chinese Yuan) | | | | GDP per capita  (Chinese Yuan) |
|  |  | Total | Primary Industry | Secondary Industry | Tertiary Industry |  |
| 1 | Yulin | 249.188 | 14.369 | 152.368 | 82.451 | 73453 |
| 2 | Xi'an | 580.12 | 22.02 | 212.629 | 345.471 | 66938 |
| 3 | Yan'an | 119.827 | 11.088 | 72.479 | 36.26 | 53908 |
| 4 | Baoji | 178.763 | 16.513 | 114.143 | 48.107 | 47565 |
| 5 | Xianyang | 215.292 | 32.878 | 123.041 | 59.373 | 43365 |
| 6 | Tongchuan | 30.716 | 2.276 | 17.031 | 11.409 | 36322 |
| 7 | Hanzhong | 105.961 | 19.153 | 45.902 | 40.906 | 30849 |
| 8 | Ankang | 75.505 | 9.606 | 40.339 | 25.56 | 28536 |
| 9 | Weinan | 143.041 | 21.392 | 69.77 | 51.879 | 26729 |
| 10 | Shangluo | 61.852 | 9.175 | 31.86 | 20.817 | 26274 |
| **Ranking of socioeconomic status of each municipal region in Yunnan Province** | | | | | | |
| Rank of GDP per capita | Municipal regions | Gross domestic products (billions Chinese Yuan) | | | | GDP per capita (Chinese Yuan) |
|  |  | Total | Primary Industry | Secondary Industry | Tertiary Industry |  |
| 1 | Kunming | 396.801 | 18.81 | 158.638 | 219.353 | 59656 |
| 2 | Yuxi | 124.452 | 12.66 | 68.39 | 43.402 | 52812 |
| 3 | Diqing | 16.114 | 1.073 | 5.632 | 9.409 | 39543 |
| 4 | Xishuangbanna | 33.591 | 8.554 | 9.463 | 15.574 | 28945 |
| 5 | Chuxiong | 76.297 | 15.282 | 29.185 | 31.83 | 27942 |
| 6 | Qujing | 163.026 | 31.715 | 64.223 | 67.088 | 27045 |
| 7 | Honghe | 122.108 | 20.199 | 55.259 | 46.65 | 26345 |
| 8 | Dali | 90.01 | 19.339 | 35.584 | 35.087 | 25459 |
| 9 | Dehong | 29.232 | 7.342 | 7.178 | 14.712 | 22990 |
| 10 | Lijiang | 28.961 | 4.457 | 11.52 | 12.984 | 22670 |
| 11 | Baoshan | 55.196 | 14.197 | 19.205 | 21.794 | 21444 |
| 12 | Nujiang | 11.315 | 1.879 | 3.437 | 5.999 | 20895 |
| 13 | Lincang | 50.212 | 14.534 | 16.98 | 18.698 | 20077 |
| 14 | Pu'er | 51.401 | 14.313 | 17.888 | 19.2 | 19773 |
| 15 | Wenshan | 67.004 | 14.645 | 24.063 | 28.296 | 18612 |
| 16 | Zhaotong | 70.838 | 14.065 | 30.813 | 25.96 | 13097 |
| **Ranking of socioeconomic status of each municipal region in Hubei Province** | | | | | | |
| Rank of GDP per capita | Municipal regions | Gross domestic products (billions Chinese Yuan) | | | | GDP per capita (Chinese Yuan) |
|  |  | Total | Primary Industry | Secondary Industry | Tertiary Industry |  |
| 1 | Wuhan | 1090.56 | 35.981 | 498.154 | 556.425 | 102808 |
| 2 | Huangshi | 122.811 | 10.856 | 67.988 | 43.967 | 49964 |
| 3 | Shiyan | 130.012 | 15.748 | 63.611 | 50.653 | 38431 |
| 4 | Yichang | 338.48 | 36.14 | 198.637 | 103.703 | 82255 |
| 5 | Xiangyang | 338.212 | 40.214 | 192.292 | 105.706 | 60244 |
| 6 | Ezhou | 73.001 | 8.466 | 42.244 | 22.291 | 68901 |
| 7 | Jingmen | 138.846 | 20.086 | 72.966 | 45.794 | 47939 |
| 8 | Xiaogan | 145.72 | 25.945 | 70.576 | 49.199 | 29873 |
| 9 | Jingzhou | 159.05 | 35.301 | 69.512 | 54.237 | 27875 |
| 10 | Huanggang | 158.924 | 37.962 | 61.842 | 59.12 | 25262 |
| 11 | Xianning | 103.007 | 17.859 | 50.047 | 35.101 | 41088 |
| 12 | Suizhou | 78.526 | 13.231 | 37.62 | 27.675 | 35844 |
| 13 | Enshi | 67.081 | 14.386 | 24.442 | 28.253 | 20163 |
| 14 | Xiantao | 59.761 | 8.799 | 31.814 | 19.148 | 51741 |
| 15 | Qianjiang | 55.757 | 6.988 | 30.517 | 18.252 | 58201 |
| 16 | Tianmen | 44.01 | 7.692 | 22.146 | 14.172 | 34063 |
| 17 | Shennongjia | 2.095 | 0.196 | 0.774 | 1.125 | 27279 |
| **Ranking of socioeconomic status of each municipal region in Henan Province** | | | | | | |
| Rank of GDP per capita | Municipal regions | Gross domestic products (billions Chinese Yuan) | | | | GDP per capita (Chinese Yuan) |
|  |  | Total | Primary Industry | Secondary Industry | Tertiary Industry |  |
| 1 | Zhengzhou | 7311·52 | 150·92 | 3604·15 | 3556·45 | 77179 |
| 2 | Jiyuan | 492·54 | 21·68 | 323·98 | 146·88 | 67797 |
| 3 | San Menxia | 1251·04 | 118·47 | 727·90 | 404·67 | 55681 |
| 4 | Jiaozuo | 1926·08 | 137·10 | 1150·96 | 638·02 | 54590 |
| 5 | Luoyang | 3469·03 | 236·39 | 1695·05 | 1537·58 | 51696 |
| 6 | Xuchang | 2171·16 | 169·58 | 1280·89 | 720·69 | 50162 |
| 7 | Hebi | 715·65 | 61·85 | 468·25 | 185·55 | 44678 |
| 8 | Luohe | 992·59 | 106·41 | 624·75 | 261·43 | 37987 |
| 9 | Puyang | 1328·34 | 157·48 | 751·19 | 419·68 | 36842 |
| 10 | Anyang | 1872·35 | 204·71 | 926·81 | 740·82 | 36695 |
| 11 | Kaifeng | 1605·84 | 283·90 | 657·40 | 664·54 | 35326 |
| 12 | Xinxiang | 1975·03 | 222·77 | 982·71 | 769·55 | 34562 |
| 13 | Ping Dingshan | 1686·01 | 167·04 | 853·76 | 665·20 | 33991 |
| 14 | Xinyang | 1879·67 | 453·86 | 750·07 | 675·74 | 29351 |
| 15 | Nanyang | 2866·82 | 501·65 | 1268·72 | 1096·45 | 28653 |
| 16 | Zhu Madian | 1807·69 | 402·52 | 720·25 | 684·93 | 26032 |
| 17 | Shangqiu | 1812·16 | 376·97 | 757·24 | 677·96 | 24940 |
| 18 | Zhoukou | 2089·70 | 454·02 | 959·61 | 676·07 | 23728 |
| **Ranking of socioeconomic status of each municipal region in Shandong Province** | | | | | | |
| Rank of GDP per capita | Municipal regions | Gross domestic products (billions Chinese Yuan) | | | | GDP per capita (Chinese Yuan) |
|  |  | Total | Primary Industry | Secondary Industry | Tertiary Industry |  |
| 1 | Dongying | 3450·64 | 117·75 | 2230·61 | 1029·90 | 163938 |
| 2 | Weihai | 3001·57 | 217·14 | 1422·22 | 1227·90 | 106922 |
| 3 | Qingdao | 9300·07 | 363·98 | 4026·46 | 4452·07 | 102519 |
| 4 | Yantai | 6446·08 | 440·85 | 3323·46 | 2395·70 | 91979 |
| 5 | Zibo | 4130·24 | 144·88 | 2228·83 | 1642·08 | 89235 |
| 6 | Jinan | 6100·23 | 305·39 | 2307·00 | 3218·65 | 85919 |
| 7 | Binzhou | 2355·33 | 217·53 | 1150·17 | 920·58 | 61189 |
| 8 | Rizhao | 1670·80 | 140·60 | 813·06 | 667·03 | 58110 |
| 9 | Tai’an | 3158·39 | 269·05 | 1461·82 | 1311·95 | 56490 |
| 10 | Weifang | 5170·53 | 455·15 | 2490·75 | 1977·13 | 55824 |
| 11 | Zaozhuang | 2031·00 | 154·11 | 1070·19 | 756·69 | 52692 |
| 12 | Laiwu | 665·83 | 52·72 | 344·16 | 261·24 | 49377 |
| 13 | Jining | 4013·12 | 454·19 | 1896·13 | 1504·06 | 48529 |
| 14 | Dezhou | 2750·94 | 283·71 | 1358·00 | 1019·34 | 48062 |
| 15 | Liaocheng | 2663·62 | 316·39 | 1360·25 | 907·16 | 44743 |
| 16 | Linyi | 3763·17 | 346·49 | 1687·10 | 1590·47 | 36656 |
| 17 | Heze | 2400·96 | 270·09 | 1267·43 | 770·77 | 28350 |

^Each shaded row indicates a selected municipal region. The data are from local official statistical data, namely Shaanxi Statistical Yearbook 2016, Yunnan Statistical Yearbook 2016, Hubei Statistical Yearbook 2016, Henan Statistical Yearbook 2016, and Shandong Statistical Yearbook 2016.^

The geographical distribution of surveyed regions in mainland China are shown as the following figure:


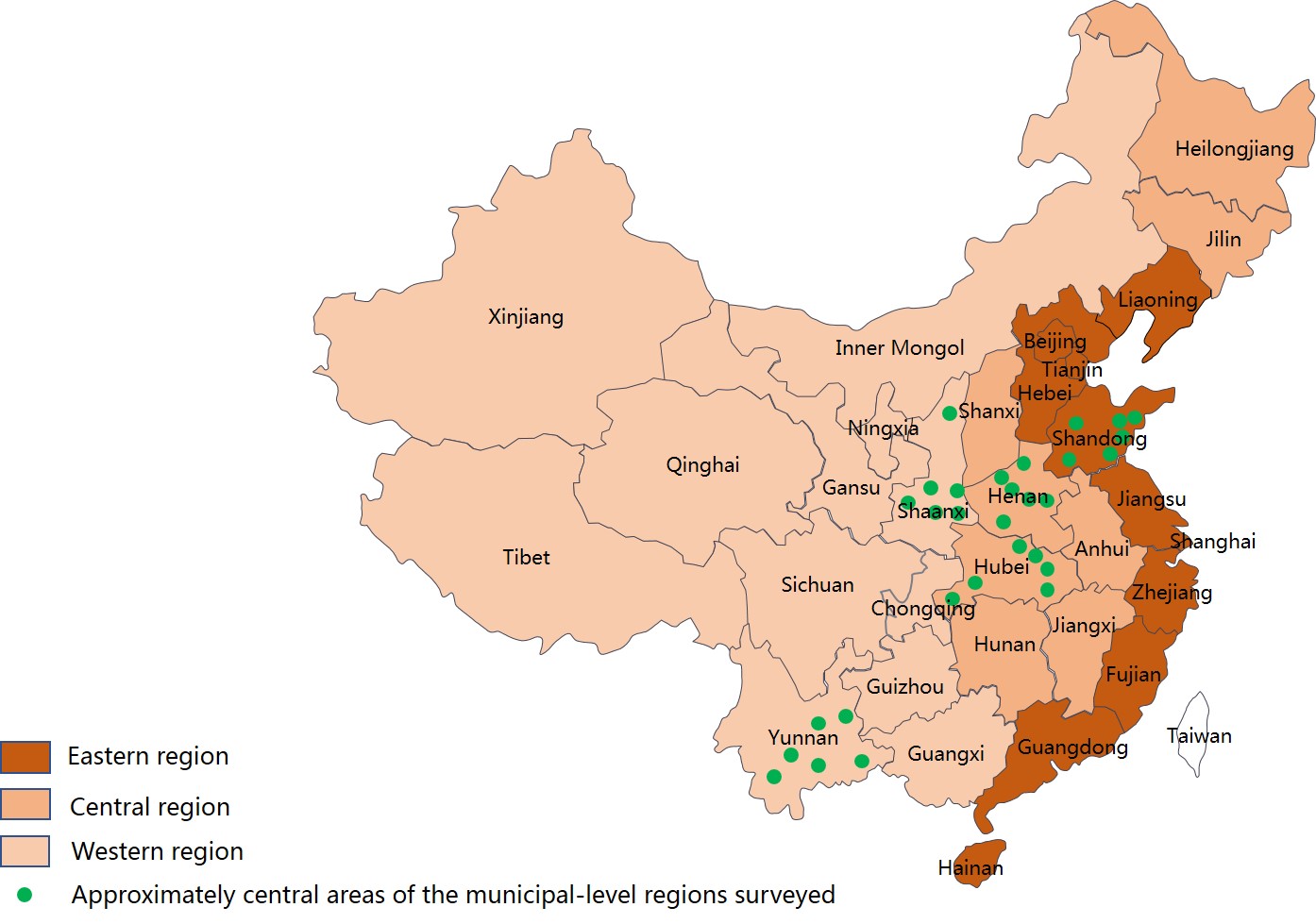


**Appendix 3.** **Information of 48 medicines surveyed**

| **Medicine** | **Strength and dosage forms** | **NEML** | **List** | **Therapeutic category** | **Disease** |
| --- | --- | --- | --- | --- | --- |
| Aciclovir | 200mg cap/tab | Yes | S | Antiviral | Acute |
| Albendazole | 200mg cap/tab(non-chewable) | Yes | S | Anthelmintic | Acute |
| Amitriptyline | 25 mg cap/tab | Yes | G | Antidepressants | Chronic |
| Amlodipine | 5mg cap/tab | Yes | S | Antihypertensive | Chronic |
| Amoxicillin | 500mg cap/tab | NO | G | Antibacterial | Acute |
| Atorvastatin | 20mg cap/tab | No | S | Hypolipemic | Chronic |
| Azithromycin | 250mg cap/tab | Yes | S | Antibacterial | Acute |
| Beclometasone inhaler | 50mcg/dose | Yes | S | Antiasthmatic | Chronic |
| Bisoprolol | 5 mg cap/tab | Yes | G | Antihypertensive | Chronic |
| Captopril | 25 mg cap/tab | Yes | G | Antihpertensive | Chronic |
| Carbamazepine | 200mg cap/tab | Yes | S | Antiepileptic | Chronic |
| Cefalexin | 250mg cap/tab | Yes | S | Antibacterial | Acute |
| Cefazolin | 1g/vial injection | Yes | S | Antibacterial | Acute |
| Ceftazidime | 1g/vial injection | Yes | S | Antibacterial | Acute |
| Ceftriaxone | 1 g/vial injection | Yes | G | Antibacterial | Acute |
| Ciprofloxacin | 500 mg cap/tab | Yes | G | Antibacterial | Acute |
| Clarithromycin | 250mg tab | Yes | S | Antibacterial | Acute |
| Co-trimoxazole | 80mg+400mg cap/tab | Yes | S | Antibacterial | Acute |
| Diazepam | 5mg cap/tab | Yes | G | Antianxiety | Chronic |
| Diclofenac | 50mg cap/tab | Yes | G | Analgesic | Acute |
| Digoxin | 0.25mg cap/tab | Yes | S | Antiarrhythmic | Chronic |
| Enalapril | 10mg cap/tab | Yes | S | Antihypertensive | Chronic |
| Erythromycin | 250mg cap/tab | Yes | S | Antibacterial | Acute |
| Fluconazole | 150mg cap/tab | Yes | S | Antifungal | Acute |
| Fluoxetine | 20mg cap/tab | No | S | Antidepressant | Chronic |
| Glargine analogue insulin | 3ml:300IU cartridge | No | S | Antidiabetic | Chronic |
| Gliclazide | 80mg cap/tab | No | S | Antidiabetic | Chronic |
| Glimepiride | 2mg tab | Yes | S | Antidiabetic | Chronic |
| Hydrochlorothiazide | 25mg cap/tab | Yes | S | Antihypertensive | Chronic |
| Isoniazid | 100mg cap/tab | Yes | S | Antibacterial | Chronic |
| Isosorbide mononitrate | 20mg caps/tab | No | S | Antianginal | Chronic |
| Ketoconazole | 200mg cap/tab | No | S | Antifungal | Acute |
| Levofloxacin | 500mg cap/tab | Yes | S | Antibacterial | Acute |
| Levothyroxine | 50mcg tab | Yes | S | Antithyroid | Chronic |
| Lisinopril | 10mg cap/tab | No | S | Antihypertensive | Chronic |
| Loratadine | 10mg cap/tab | yes | S | Antiallergic | Acute |
| Losartan | 50mg cap/tab | No | S | Antihypertensive | Chronic |
| Lovastatin | 20mg cap/tab | No | S | Hypolipemic | Chronic |
| Metformin | 500 mg cap/tab | Yes | G | Antidiabetic | Chronic |
| Metronidazole | 200mgcap/tab | Yes | S | Antibacterial | Acute |
| Nimodipine | 30mg tab | Yes | S | Antihypertensive | Chronic |
| Omeprazole | 20mg Cap/tab | Yes | G | Antiulcer | Acute |
| Phenytoin Sodium | 100mg cap/tab | Yes | S | Antiepileptic | Chronic |
| Ranitidine | 150mg cap/tab | Yes | S | Antiulcer | Acute |
| Regular human insulin | 3ml:300IU cartridge | Yes | S | Antidiabetic | Chronic |
| Rifampicin | 150mg tab | Yes | S | Antibacterial | Chronic |
| Salbutamol | 0.1 mg/dose inhaler | Yes | G | Antiasthmatic | Chronic |
| Simvastatin | 20 mg cap/tab | Yes | G | Hypolipemic | Chronic |

NEML=2012 National Essential Medicines List; G=Global list suggested by WHO/HAI; S=Supplementary list.

**Appendix 4. Details regarding surveyed pubic hospitals, private pharmacies and medicine information of the panel in Shaanxi Province, 2010-2018**

The health facility selection procedure of the panel in Shaanxi Province is same as that in the national survey. The distribution of surveyed pubic hospitals, private pharmacies in Shaanxi Province is shown as the following table:

| Area | **2010 survey** | | **2012 survey** | | **2014 survey** | | **2018 survey** | |
| --- | --- | --- | --- | --- | --- | --- | --- | --- |
|  | Public hospitals | Private pharmacies | Public hospitals | Private pharmacies | Public hospitals | Private pharmacies | Public hospitals | Private pharmacies |
| Xi’an | 4/3/1 | 7 | 6/4/2 | 12 | 6/4/2 | 12 | 6/4/1 | 7 |
| Yulin | 4/3/1 | 8 | 6/4/2 | 12 | 6/4/2 | 12 | 6/2/1 | 10 |
| Xianyang | 4/3/1 | 5 | 6/4/2 | 12 | 6/4/2 | 12 | 6/4/1 | 9 |
| Baoji | 4/4/1 | 5 | 6/4/2 | 12 | 4/4/2 | 12 | 6/4/1 | 12 |
| Weinan | 4/4/1 | 6 | 6/4/2 | 12 | 4/4/2 | 12 | 6/4/1 | 9 |
| Shangluo | 4/3/1 | 5 | 6/4/2 | 12 | 6/4/2 | 12 | 6/4/1 | 8 |
| **Subtotal** | 24/20/6 (50) | 36 | 36/24/12  (72) | 72 | 32/24/12 (68) | 72 | 36/22/6 (64) | 55 |

^The number of primary, secondary and tertiary hospitals surveyed are separated by slash. The number inside those parentheses are total number of surveyed hospitals.^

There were 31medicines surveyed in the four years. Of these medicines, 11 were from the WHO/HAI core list and 20 were supplementary medicines; 25 were on the NEML and 6 were not; 15 medicines treat acute disorders, whereas 16 treat chronic disorders. The details are showing as following table:

| **Medicine** | **Strength and Dosage forms** | **NEML** | **List** | **Therapeutic category** |
| --- | --- | --- | --- | --- |
| Aciclovir | 200mg cap/tab | Yes | S | Antiviral |
| Albendazole | 200mg cap/tab(non-chewable) | Yes | S | Anthelmintic |
| Amitriptyline | 25 mg cap/tab | Yes | G | Antidepressants |
| Amlodipine | 5mg cap/tab | Yes | S | Antihypertensive |
| Amoxicillin | 500mg cap/tab | NO | G | Antibacterial |
| Azithromycin | 250mg cap/tab | Yes | S | Antibacterial |
| Beclometasone inhaler | 50mcg/dose | Yes | S | Anti-asthmatic |
| Captopril | 25 mg cap/tab | Yes | G | Antihypertensive |
| Cefalexin | 250mg cap/tab | Yes | S | Antibacterial |
| Ceftriaxone | 1 g/vial injection | Yes | G | Antibacterial |
| Ciprofloxacin | 500 mg cap/tab | Yes | G | Antibacterial |
| Diazepam | 5mg cap/tab | Yes | G | Antianxiety |
| Diclofenac | 50mg cap/tab | Yes | G | Analgesic |
| Digoxin | 0.25mg cap/tab | Yes | S | Antiarrhythmic |
| Enalapril | 10mg cap/tab | Yes | S | Antihypertensive |
| Erythromycin | 250mg cap/tab | Yes | S | Antibacterial |
| Fluconazole | 150mg cap/tab | Yes | S | Antifungal |
| Fluoxetine | 20mg cap/tab | No | S | Antidepressant |
| Hydrochlorothiazide | 25mg cap/tab | Yes | S | Antihypertensive |
| Ketoconazole | 200mg cap/tab | No | S | Antifungal |
| Lisinopril | 10mg cap/tab | No | S | Antihypertensive |
| Loratadine | 10mg cap/tab | yes | S | Antiallergic |
| Losartan | 50mg cap/tab | No | S | Antihypertensive |
| Lovastatin | 20mg cap/tab | No | S | Hypolipidemic |
| Metformin | 500 mg cap/tab | Yes | G | Antidiabetic |
| Metronidazole | 200mgcap/tab | Yes | S | Antibacterial |
| Omeprazole | 20mg Cap/tab | Yes | G | Antiulcer |
| Ranitidine | 150mg cap/tab | Yes | S | Antiulcer |
| Rifampicin | 150mg tab | Yes | S | Antibacterial |
| Salbutamol | 0.1 mg/dose inhaler | Yes | G | Antiasthmatic |
| Simvastatin | 20 mg cap/tab | Yes | G | Hypolipidemic |

NEML=2012 National Essential Medicines List; G=Global list; S=Supplementary list.

**Appendix 5. Availability of 48 medicines in public sector hospitals and private retail pharmacies in surveyed provinces**

| **Availability in Shandong Province (%)** | | | | |
| --- | --- | --- | --- | --- |
| **Medicine** | **Public sector** | | **Private sector** | |
|  | **OB** | **Generic** | **OB** | **Generic** |
| Aciclovir | 0.0% | 23.3% | 0.0% | 34.8% |
| Albendazole | 8.3% | 15.8% | 71.7% | 26.1% |
| Amitriptyline | 0.0% | 5.8% | 2.2% | 10.9% |
| Amlodipine | 20.0% | 10.0% | 39.1% | 45.7% |
| Amoxicillin | 9.2% | 5.0% | 30.4% | 58.7% |
| Atorvastatin | 14.2% | 11.7% | 56.5% | 43.5% |
| Azithromycin | 3.3% | 30.8% | 10.9% | 82.6% |
| Beclometasone inhaler | 0.0% | 1.7% | 0.0% | 4.3% |
| Bisoprolol | 8.3% | 8.3% | 26.1% | 37.0% |
| Captopril | 1.7% | 23.3% | 0.0% | 69.6% |
| Carbamazepine | 5.0% | 7.5% | 39.1% | 8.7% |
| Cefalexin | 0.0% | 13.3% | 0.0% | 73.9% |
| Cefazolin | 0.0% | 13.3% | 0.0% | 0.0% |
| Ceftazidime | 0.0% | 16.7% | 0.0% | 0.0% |
| Ceftriaxone injection | 14.2% | 23.3% | 0.0% | 0.0% |
| Ciprofloxacin | 0.0% | 0.0% | 0.0% | 2.2% |
| Clarithromycin | 0.0% | 9.2% | 6.5% | 13.0% |
| Co-trimoxazole | 0.0% | 3.3% | 0.0% | 45.7% |
| Diazepam | 0.0% | 10.8% | 0.0% | 2.2% |
| Diclofenac | 0.0% | 3.3% | 2.2% | 32.6% |
| Digoxin | 0.8% | 25.0% | 0.0% | 39.1% |
| Enalapril | 0.0% | 21.7% | 15.2% | 58.7% |
| Erythromycin | 0.0% | 10.0% | 0.0% | 0.0% |
| Fluconazole | 3.3% | 0.0% | 15.2% | 19.6% |
| Fluoxetine | 7.5% | 5.0% | 8.7% | 2.2% |
| Glargine analogue insulin | 17.5% | 4.2% | 8.7% | 0.0% |
| Gliclazide | 1.7% | 5.8% | 28.3% | 37.0% |
| Glimepiride | 11.7% | 25.8% | 23.9% | 67.4% |
| Hydrochlorothiazide | 0.0% | 29.2% | 2.2% | 63.0% |
| Isoniazid | 0.0% | 7.5% | 0.0% | 6.5% |
| Isosorbide mononitrate | 0.8% | 27.5% | 4.3% | 71.7% |
| Ketoconazole | 0.0% | 0.0% | 0.0% | 2.2% |
| Levofloxacin | 0.0% | 5.8% | 0.0% | 37.0% |
| Levothyroxine | 18.3% | 0.8% | 69.6% | 4.3% |
| Lisinopril | 0.0% | 2.5% | 0.0% | 15.2% |
| Loratadine | 5.8% | 25.0% | 30.4% | 69.6% |
| Losartan | 3.3% | 5.8% | 17.4% | 10.9% |
| Lovastatin | 0.0% | 3.3% | 0.0% | 13.0% |
| Metformin | 12.5% | 14.2% | 47.8% | 21.7% |
| Metronidazole | 0.0% | 26.7% | 0.0% | 63.0% |
| Nimodipine | 7.5% | 20.8% | 30.4% | 43.5% |
| Omeprazole | 3.3% | 25.0% | 23.9% | 76.1% |
| Phenytoin | 0.0% | 12.5% | 0.0% | 21.7% |
| Ranitidine | 0.0% | 15.0% | 0.0% | 69.6% |
| Regular human insulin | 7.5% | 3.3% | 4.3% | 0.0% |
| Rifampicin | 0.0% | 6.7% | 0.0% | 13.0% |
| Salbutamol inhaler | 13.3% | 10.8% | 21.7% | 52.2% |
| Simvastatin | 6.7% | 12.5% | 47.8% | 43.5% |
| **All medicines** | **4.3%** | **12.3%** | **14.3%** | **31.5%** |
| **Availability in Hubei Province (%)** | | | | |
| **Medicine** | **Public sector** | | **Private sector** | |
|  | **OB** | **Generic** | **OB** | **Generic** |
| Aciclovir | 0.0% | 12.9% | 0.0% | 12.1% |
| Albendazole | 50.0% | 6.5% | 77.6% | 13.8% |
| Amitriptyline | 1.6% | 19.4% | 5.2% | 8.6% |
| Amlodipine | 33.9% | 41.9% | 46.6% | 53.4% |
| Amoxicillin | 6.5% | 9.7% | 25.9% | 63.8% |
| Atorvastatin | 40.3% | 14.5% | 53.4% | 25.9% |
| Azithromycin | 1.6% | 69.4% | 8.6% | 74.1% |
| Beclometasone inhaler | 0.0% | 8.1% | 0.0% | 0.0% |
| Bisoprolol | 32.3% | 16.1% | 43.1% | 24.1% |
| Captopril | 0.0% | 62.9% | 1.7% | 77.6% |
| Carbamazepine | 19.4% | 0.0% | 31.0% | 1.7% |
| Cefalexin | 0.0% | 12.9% | 0.0% | 12.1% |
| Cefazolin | 0.0% | 17.7% | 0.0% | 0.0% |
| Ceftazidime | 1.6% | 59.7% | 0.0% | 1.7% |
| Ceftriaxone injection | 8.1% | 46.8% | 0.0% | 5.2% |
| Ciprofloxacin | 0.0% | 1.6% | 0.0% | 0.0% |
| Clarithromycin | 0.0% | 17.7% | 3.4% | 27.6% |
| Co-trimoxazole | 0.0% | 8.1% | 0.0% | 19.0% |
| Diazepam | 0.0% | 3.2% | 0.0% | 1.7% |
| Diclofenac | 0.0% | 9.7% | 5.2% | 12.1% |
| Digoxin | 4.8% | 50.0% | 3.4% | 39.7% |
| Enalapril | 4.8% | 19.4% | 0.0% | 60.3% |
| Erythromycin | 0.0% | 14.5% | 0.0% | 5.2% |
| Fluconazole | 3.2% | 8.1% | 1.7% | 24.1% |
| Fluoxetine | 8.1% | 3.2% | 3.4% | 1.7% |
| Glargine analogue insulin | 48.4% | 9.7% | 17.2% | 1.7% |
| Gliclazide | 12.9% | 14.5% | 36.2% | 36.2% |
| Glimepiride | 25.8% | 56.5% | 25.9% | 70.7% |
| Hydrochlorothiazide | 0.0% | 61.3% | 0.0% | 44.8% |
| Isoniazid | 0.0% | 30.6% | 0.0% | 29.3% |
| Isosorbide mononitrate | 0.0% | 22.6% | 0.0% | 41.4% |
| Ketoconazole | 0.0% | 3.2% | 1.7% | 0.0% |
| Levofloxacin | 0.0% | 4.8% | 0.0% | 17.2% |
| Levothyroxine | 21.0% | 11.3% | 58.6% | 0.0% |
| Lisinopril | 0.0% | 12.9% | 0.0% | 20.7% |
| Loratadine | 12.9% | 37.1% | 48.3% | 82.8% |
| Losartan | 14.5% | 11.3% | 25.9% | 1.7% |
| Lovastatin | 0.0% | 9.7% | 1.7% | 17.2% |
| Metformin | 35.5% | 35.5% | 46.6% | 20.7% |
| Metronidazole | 0.0% | 71.0% | 0.0% | 69.0% |
| Nimodipine | 14.5% | 4.8% | 10.3% | 1.7% |
| Omeprazole | 4.8% | 74.2% | 31.0% | 79.3% |
| Phenytoin | 0.0% | 12.9% | 0.0% | 3.4% |
| Ranitidine | 1.6% | 25.8% | 0.0% | 43.1% |
| Regular human insulin | 6.5% | 37.1% | 8.6% | 1.7% |
| Rifampicin | 0.0% | 21.0% | 0.0% | 32.8% |
| Salbutamol inhaler | 22.6% | 25.8% | 10.3% | 51.7% |
| Simvastatin | 8.1% | 32.3% | 15.5% | 29.3% |
| **All medicines** | **9.3%** | **24.2%** | **13.5%** | **26.3%** |
| **Availability in Henan Province (%)** | | | | |
| **Medicine** | **Public sector** | | **Private sector** | |
|  | **OB** | **Generic** | **OB** | **Generic** |
| Aciclovir | 0.0% | 37.0% | 0.0% | 43.2% |
| Albendazole | 22.2% | 7.4% | 86.4% | 47.7% |
| Amitriptyline | 0.0% | 44.4% | 0.0% | 59.1% |
| Amlodipine | 37.0% | 38.9% | 68.2% | 47.7% |
| Amoxicillin | 7.4% | 29.6% | 40.9% | 43.2% |
| Atorvastatin | 42.6% | 14.8% | 77.3% | 38.6% |
| Azithromycin | 5.6% | 64.8% | 29.5% | 77.3% |
| Beclometasone inhaler | 0.0% | 3.7% | 4.5% | 15.9% |
| Bisoprolol | 16.7% | 24.1% | 31.8% | 43.2% |
| Captopril | 0.0% | 75.9% | 2.3% | 68.2% |
| Carbamazepine | 9.3% | 20.4% | 45.5% | 20.5% |
| Cefalexin | 0.0% | 42.6% | 4.5% | 47.7% |
| Cefazolin | 0.0% | 44.4% | 0.0% | 18.2% |
| Ceftazidime | 5.6% | 48.1% | 2.3% | 25.0% |
| Ceftriaxone injection | 9.3% | 61.1% | 13.6% | 31.8% |
| Ciprofloxacin | 0.0% | 5.6% | 0.0% | 13.6% |
| Clarithromycin | 1.9% | 3.7% | 4.5% | 38.6% |
| Co-trimoxazole | 0.0% | 31.5% | 0.0% | 54.5% |
| Diazepam | 0.0% | 13.0% | 0.0% | 4.5% |
| Diclofenac | 0.0% | 33.3% | 38.6% | 18.2% |
| Digoxin | 0.0% | 75.9% | 0.0% | 47.7% |
| Enalapril | 0.0% | 40.7% | 0.0% | 63.6% |
| Erythromycin | 0.0% | 9.3% | 0.0% | 18.2% |
| Fluconazole | 7.4% | 9.3% | 25.0% | 25.0% |
| Fluoxetine | 7.4% | 3.7% | 20.5% | 9.1% |
| Glargine analogue insulin | 33.3% | 13.0% | 34.1% | 27.3% |
| Gliclazide | 11.1% | 37.0% | 63.6% | 50.0% |
| Glimepiride | 22.2% | 63.0% | 47.7% | 59.1% |
| Hydrochlorothiazide | 0.0% | 64.8% | 6.8% | 50.0% |
| Isoniazid | 0.0% | 22.2% | 0.0% | 43.2% |
| Isosorbide mononitrate | 1.9% | 42.6% | 13.6% | 54.5% |
| Ketoconazole | 0.0% | 1.9% | 13.6% | 0.0% |
| Levofloxacin | 0.0% | 20.4% | 0.0% | 43.2% |
| Levothyroxine | 29.6% | 11.1% | 70.5% | 4.5% |
| Lisinopril | 0.0% | 5.6% | 0.0% | 22.7% |
| Loratadine | 13.0% | 53.7% | 50.0% | 65.9% |
| Losartan | 18.5% | 16.7% | 45.5% | 15.9% |
| Lovastatin | 0.0% | 18.5% | 0.0% | 43.2% |
| Metformin | 31.5% | 37.0% | 68.2% | 27.3% |
| Metronidazole | 0.0% | 81.5% | 0.0% | 70.5% |
| Nimodipine | 3.7% | 37.0% | 27.3% | 43.2% |
| Omeprazole | 11.1% | 79.6% | 59.1% | 68.2% |
| Phenytoin | 0.0% | 35.2% | 0.0% | 59.1% |
| Ranitidine | 0.0% | 59.3% | 0.0% | 72.7% |
| Regular human insulin | 24.1% | 35.2% | 43.2% | 20.5% |
| Rifampicin | 0.0% | 11.1% | 0.0% | 38.6% |
| Salbutamol inhaler | 24.1% | 3.7% | 11.4% | 43.2% |
| Simvastatin | 13.0% | 44.4% | 47.7% | 54.5% |
| **All medicines** | **8.5%** | **32.9%** | **22.9%** | **39.5%** |
| **Availability in Shaanxi Province (%)** | | | | |
| **Medicine** | **Public sector** | | **Private sector** | |
|  | **OB** | **Generic** | **OB** | **Generic** |
| Aciclovir | 0.0% | 12.5% | 0.0% | 20.0% |
| Albendazole | 14.1% | 6.3% | 78.2% | 12.7% |
| Amitriptyline | 0.0% | 10.9% | 0.0% | 5.5% |
| Amlodipine | 37.5% | 15.6% | 65.5% | 63.6% |
| Amoxicillin | 14.1% | 10.9% | 29.1% | 63.6% |
| Atorvastatin | 46.9% | 26.6% | 50.9% | 67.3% |
| Azithromycin | 9.4% | 42.2% | 18.2% | 69.1% |
| Beclometasone inhaler | 0.0% | 1.6% | 0.0% | 1.8% |
| Bisoprolol | 9.4% | 14.1% | 29.1% | 45.5% |
| Captopril | 0.0% | 31.3% | 3.6% | 70.9% |
| Carbamazepine | 3.1% | 1.6% | 27.3% | 5.5% |
| Cefalexin | 0.0% | 6.3% | 0.0% | 18.2% |
| Cefazolin | 0.0% | 10.9% | 0.0% | 0.0% |
| Ceftazidime | 6.3% | 32.8% | 0.0% | 3.6% |
| Ceftriaxone injection | 18.8% | 53.1% | 0.0% | 18.2% |
| Ciprofloxacin | 0.0% | 1.6% | 0.0% | 3.6% |
| Clarithromycin | 1.6% | 20.3% | 0.0% | 36.4% |
| Co-trimoxazole | 0.0% | 17.2% | 0.0% | 36.4% |
| Diazepam | 0.0% | 3.1% | 0.0% | 0.0% |
| Diclofenac | 0.0% | 21.9% | 0.0% | 36.4% |
| Digoxin | 0.0% | 39.1% | 0.0% | 56.4% |
| Enalapril | 0.0% | 67.2% | 0.0% | 83.6% |
| Erythromycin | 0.0% | 3.1% | 0.0% | 10.9% |
| Fluconazole | 1.6% | 9.4% | 0.0% | 25.5% |
| Fluoxetine | 1.6% | 3.1% | 7.3% | 1.8% |
| Glargine analogue insulin | 34.4% | 17.2% | 34.5% | 12.7% |
| Gliclazide | 28.1% | 20.3% | 49.1% | 47.3% |
| Glimepiride | 20.3% | 15.6% | 34.5% | 63.6% |
| Hydrochlorothiazide | 0.0% | 57.8% | 3.6% | 54.5% |
| Isoniazid | 0.0% | 20.3% | 0.0% | 40.0% |
| Isosorbide mononitrate | 1.6% | 34.4% | 1.8% | 56.4% |
| Ketoconazole | 0.0% | 0.0% | 0.0% | 0.0% |
| Levofloxacin | 0.0% | 6.3% | 0.0% | 30.9% |
| Levothyroxine | 20.3% | 0.0% | 34.5% | 0.0% |
| Lisinopril | 0.0% | 7.8% | 0.0% | 25.5% |
| Loratadine | 4.7% | 50.0% | 36.4% | 74.5% |
| Losartan | 12.5% | 12.5% | 38.2% | 40.0% |
| Lovastatin | 0.0% | 7.8% | 1.8% | 21.8% |
| Metformin | 40.6% | 25.0% | 60.0% | 38.2% |
| Metronidazole | 0.0% | 75.0% | 0.0% | 76.4% |
| Nimodipine | 7.8% | 6.3% | 21.8% | 5.5% |
| Omeprazole | 12.5% | 70.3% | 36.4% | 78.2% |
| Phenytoin | 0.0% | 1.6% | 0.0% | 7.3% |
| Ranitidine | 0.0% | 25.0% | 0.0% | 61.8% |
| Regular human insulin | 34.4% | 9.4% | 20.0% | 9.1% |
| Rifampicin | 0.0% | 10.9% | 0.0% | 40.0% |
| Salbutamol inhaler | 21.9% | 20.3% | 32.7% | 23.6% |
| Simvastatin | 21.9% | 10.9% | 36.4% | 60.0% |
| **All medicines** | **8.9%** | **20.1%** | **15.6%** | **33.8%** |
| **Availability in Yunnan Province (%)** | | | | |
| **Medicine** | **Public sector** | | **Private sector** | |
|  | **OB** | **Generic** | **OB** | **Generic** |
| Aciclovir | 0.0% | 14.2% | 0.0% | 27.8% |
| Albendazole | 15.0% | 13.3% | 94.4% | 36.1% |
| Amitriptyline | 0.0% | 10.0% | 0.0% | 13.9% |
| Amlodipine | 21.7% | 27.5% | 88.9% | 63.9% |
| Amoxicillin | 3.3% | 9.2% | 94.4% | 52.8% |
| Atorvastatin | 21.7% | 11.7% | 94.4% | 47.2% |
| Azithromycin | 5.0% | 9.2% | 88.9% | 63.9% |
| Beclometasone inhaler | 0.0% | 4.2% | 0.0% | 0.0% |
| Bisoprolol | 14.2% | 8.3% | 86.1% | 13.9% |
| Captopril | 0.8% | 11.7% | 0.0% | 44.4% |
| Carbamazepine | 7.5% | 5.0% | 83.3% | 0.0% |
| Cefalexin | 0.0% | 0.0% | 16.7% | 22.2% |
| Cefazolin | 0.0% | 13.3% | 0.0% | 0.0% |
| Ceftazidime | 0.8% | 22.5% | 0.0% | 0.0% |
| Ceftriaxone injection | 5.8% | 22.5% | 0.0% | 0.0% |
| Ciprofloxacin | 0.0% | 0.8% | 0.0% | 2.8% |
| Clarithromycin | 1.7% | 5.0% | 83.3% | 33.3% |
| Co-trimoxazole | 0.0% | 9.2% | 0.0% | 33.3% |
| Diazepam | 0.0% | 2.5% | 0.0% | 5.6% |
| Diclofenac | 0.0% | 20.0% | 2.8% | 61.1% |
| Digoxin | 0.0% | 18.3% | 0.0% | 47.2% |
| Enalapril | 0.0% | 23.3% | 0.0% | 61.1% |
| Erythromycin | 0.0% | 5.8% | 0.0% | 8.3% |
| Fluconazole | 1.7% | 9.2% | 72.2% | 25.0% |
| Fluoxetine | 8.3% | 2.5% | 86.1% | 11.1% |
| Glargine analogue insulin | 19.2% | 2.5% | 88.9% | 16.7% |
| Gliclazide | 3.3% | 14.2% | 44.4% | 55.6% |
| Glimepiride | 9.2% | 16.7% | 91.7% | 58.3% |
| Hydrochlorothiazide | 0.8% | 21.7% | 8.3% | 44.4% |
| Isoniazid | 0.0% | 13.3% | 0.0% | 13.9% |
| Isosorbide mononitrate | 5.8% | 11.7% | 75.0% | 47.2% |
| Ketoconazole | 0.8% | 0.8% | 69.4% | 2.8% |
| Levofloxacin | 0.0% | 9.2% | 0.0% | 19.4% |
| Levothyroxine | 13.3% | 5.0% | 97.2% | 5.6% |
| Lisinopril | 0.8% | 2.5% | 80.6% | 16.7% |
| Loratadine | 5.0% | 26.7% | 91.7% | 61.1% |
| Losartan | 8.3% | 8.3% | 97.2% | 38.9% |
| Lovastatin | 0.0% | 13.3% | 0.0% | 30.6% |
| Metformin | 9.2% | 23.3% | 94.4% | 44.4% |
| Metronidazole | 0.0% | 33.3% | 0.0% | 83.3% |
| Nimodipine | 10.8% | 1.7% | 94.4% | 5.6% |
| Omeprazole | 7.5% | 26.7% | 88.9% | 72.2% |
| Phenytoin | 0.0% | 6.7% | 0.0% | 44.4% |
| Ranitidine | 0.0% | 15.0% | 0.0% | 80.6% |
| Regular human insulin | 8.3% | 8.3% | 11.1% | 11.1% |
| Rifampicin | 0.0% | 13.3% | 0.0% | 38.9% |
| Salbutamol inhaler | 17.5% | 0.8% | 86.1% | 33.3% |
| Simvastatin | 12.5% | 10.0% | 88.9% | 41.7% |
| **All medicines** | **5.0%** | **11.8%** | **43.8%** | **32.1%** |

**Appendix 6. The median price (RMB) of glargine analogue insulin (3ml:300IU) in five Provinces**

|  | **Shandong** | **Hubei** | **Henan** | **Shaanxi** | **Yunnan** |
| --- | --- | --- | --- | --- | --- |
| LPG in Public | 146.8 | 145.5 | 145.5 | 154.8 | 169.1 |
| OB in public | 170.6 | 184.6 | 184.6 | 206.1 | 216.5 |
| LPG in private | / | / | 145 | 154.8 | 175.8 |
| OB in private | 178.2 | 214.0 | 184.6 | 215.0 | 227.3 |

“/”: no median price because that the medicine was not available in at least three private facilities.

**Appendix 7. Average availability of 31medicines in all public sector hospitals and in 20 commonly surveyed hospitals in Shaanxi Province**

| **Medicines categories** | | **Average availability in all public sector hospitals (%)** | | | | **Average availability in commonly surveyed public hospitals (%)** | | | |
| --- | --- | --- | --- | --- | --- | --- | --- | --- | --- |
| **All hospitals** | | **2010**  **(n=50)** | **2012**  **(n=72)** | **2014**  **(n=68)** | **2018**  **(n=64)** | **2010**  **(n=24)** | **2012**  **(n=24)** | **2014**  **(n=24)** | **2018**  **(n=24)** |
| **Generics** | All(31) | 28.1 | 23.0 | 23.8 | 23.0 | 33.5 | 25.8 | 27.9 | 25.8 |
|  | NEML(25) | 33.2 | 26.6 | 26.3 | 26.8 | 38.7 | 29.3 | 31.0 | 29.3 |
|  | Non-NEML(6) | 7.0 | 8.1 | 13.0 | 7.0 | 11.8 | 11.1 | 14.8 | 11.3 |
|  | Global(11) | 30.6 | 22.7 | 23.2 | 23.6 | 35.2 | 24.6 | 22.7 | 24.7 |
|  | Supplementary(20) | 26.8 | 23.2 | 24.1 | 22.7 | 32.5 | 26.5 | 30.7 | 26.4 |
|  | Acute(15) | 29.7 | 23.8 | 22.7 | 25.8 | 32.2 | 24.2 | 26.1 | 23.2 |
|  | Chronic(16) | 26.6 | 22.3 | 24.7 | 20.3 | 34.6 | 27.3 | 29.5 | 28.3 |
| **OBs** | All(31) | 8.6 | 8.3 | 7.6 | 6.8 | 13.4 | 13.2 | 11.9 | 11.6 |
|  | NEML(25) | 8.4 | 8.6 | 7.9 | 7.3 | 13.3 | 14.0 | 12.8 | 12.2 |
|  | Non-NEML(6) | 9.3 | 7.2 | 6.1 | 4.7 | 13.9 | 9.7 | 7.9 | 9.3 |
|  | Global(11) | 9.8 | 11.2 | 11.9 | 11.8 | 16.7 | 18.6 | 15.2 | 20.7 |
|  | Supplementary(20) | 7.9 | 6.7 | 5.2 | 4.1 | 11.7 | 10.2 | 10.1 | 6.6 |
|  | Acute(15) | 8.1 | 7.4 | 6.2 | 5.0 | 12.5 | 11.9 | 8.9 | 8.3 |
|  | Chronic(16) | 9.0 | 9.1 | 8.8 | 8.5 | 14.3 | 14.3 | 14.7 | 14.8 |

**Appendix 8. Median patient prices of 31 medicines in all and in commonly surveyed hospitals**

| Medicines categories | Patient prices in all public sector hospitals (Yuan) | | | | | Medicines categories | Patient prices in commonly surveyed hospitals (Yuan) | | | | |
| --- | --- | --- | --- | --- | --- | --- | --- | --- | --- | --- | --- |
| All hospitals | 2010 | 2012 | 2014 | 2018 | 2018-2010 | All hospitals | 2010 | 2012 | 2014 | 2018 | 2018-2010 |
| **LPGs*** All(20) | 0.47 | 0.41 | 0.47 | 0.22 | -21.4[-41.4,12.4] | **LPGs*** All(12) | 0.16 | 0.16 | 0.14 | 0.13 | -10.4[-48.7,16.0] |
| NEML(16) | 0.28 | 0.20 | 0.33 | 0.17 | -14.1[-39.3,16.9] | NEML(11) | 0.16 | 0.16 | 0.14 | 0.13 | -10.4[-36.3,19.3] |
| Non-NEML(3) | 1.27 | 0.96 | 0.82 | 0.81 | -36.2[-51.9,-27.9] | Non-NEML(1) | ----- | ----- | ----- | ----- | ----- |
| Global(7) | 0.47 | 0.41 | 0.42 | 0.17 | -26.5[-55.7,1.1] | Global(4) | 0.33 | 0.62 | 0.28 | 0.38 | -21.9[-32.9,-7.3] |
| Supplementary(12) | 0.56 | 0.55 | 0.48 | 0.36 | -20.5[-36.5,16.9] | Supplementary(8) | 0.53 | 0.56 | 0.30 | 0.30 | -4.7[-48.7,16.0] |
| Acute(9) | 0.47 | 0.41 | 0.50 | 0.22 | -33.5[-47.9,20.7] | Acute(6) | 0.71 | 1.12 | 0.47 | 0.56 | -6.9[-36.8,25.6] |
| Chronic(10) | 0.54 | 0.50 | 0.31 | 0.33 | -13.2[-33.8,8.8] | Chronic(6) | 0.15 | 0.16 | 0.14 | 0.13 | -21.9[-42.4,-12.7] |
| **OBs** All(9) | 4.6 | 3.3 | 3.2 | 3.17 | -22.4[-34.8,-20.0] | **OBs**  All(7) | 3.70 | 3.29 | 3.03 | 2.49 | -34.5[-35.8,-33.3] |
| NEML(8) | 4.3 | 3.2 | 3.1 | 3.1 | -22.5[-35.7,-19.7] | NEML(6) | 2.66 | 2.32 | 2.15 | 1.76 | -34.7[-35.7, -33.7] |
| Non-NEML(1) | ----- | ----- | ----- | ----- | ----- | Non-NEML(1) | ----- | ----- | ----- | ----- | ----- |
| Global(5) | 4.6 | 3.3 | 3.0 | 3.0 | -34.8[-38.5,-22.6] | Global(4) | 2.66 | 2.32 | 2.15 | 1.76 | -33.7[-34.1,-31.0] |
| Supplementary(4) | 4.9 | 4.0 | 4.0 | 3.9 | -19.3[-20.2,17.4] | Supplementary(3) | 5.89 | 5.01 | 4.07 | 3.90 | -35.8[-39.1,-35.0] |
| Acute(4) | 9.1 | 8.1 | 7.5 | 7.1 | -20.6[-26.7,-17.4] | Acute(2) | ----- | ----- | ----- | ----- | ----- |
| Chronic(5) | 4.6 | 3.3 | 3.0 | 3.0 | -22.4[-34.8,-21.0] | Chronic(5) | 3.70 | 3.29 | 3.03 | 2.49 | -34.7[-35.8,-33.7] |

*: we excluded the price of LPG Digoxin as it’s an abnormal value. During 2015 to 2017, the price of generic digoxin increased more than 10 times because of shortage.
